# Assessment of lipophilicity of newly synthesized celecoxib analogues using reversed-phase HPLC

**DOI:** 10.1186/s13065-019-0607-6

**Published:** 2019-07-09

**Authors:** Heba Elmansi, Jenny Jeehan Nasr, Azza H. Rageh, Mohamed I. El-Awady, Ghada S. Hassan, Hatem A. Abdel-Aziz, Fathalla Belal

**Affiliations:** 1 Department of Pharmaceutical Analytical Chemistry, Faculty of Pharmacy, Mansoura University, Mansoura, 35516 Egypt; 2 Department of Pharmaceutical Analytical Chemistry, Faculty of Pharmacy, Assiut University, Assiut, 71526 Egypt; 3 Department of Medicinal Chemistry, Faculty of Pharmacy, Mansoura University, Mansoura, 35516 Egypt; 4 Department of Applied Organic Chemistry, National Research Centre, Dokki, 12622 Egypt

**Keywords:** Lipophilicity estimation, Celecoxib analogues, HPLC, Lipophilicity chromatographic index, log *k*_w_

## Abstract

**Background:**

Lipophilicity is a physicochemical property of an essential importance in medicinal chemistry; therefore, fast and reliable measurement of lipophilicity will affect greatly the drug discovery process.

**Results:**

A series of *N*-benzenesulfonamide-1*H*-pyrazoles, oximes and hydrazones as celecoxib analogues was investigated with regard to their retention behavior using reversed-phase high performance liquid chromatography (RP-HPLC). The mobile phases employed for this study consist of a mixture of water and methanol in different proportions. In addition, the stationary phase utilized for this separation was C_18_ silanized silica gel and using 200 nm as a detection wavelength. The retention behavior of the investigated compounds was determined based on practical determination of log *k* at different concentrations of methanol (as an organic modifier) in the mobile phase ranging from 60 to 80%. It was observed that the retention of these compounds (expressed as log *k*) decreased in a linear manner with increasing the concentration of methanol. High correlation coefficients (more than 0.90 in most cases) were obtained for the relationship between the volume fraction of the organic solvent and the retention values represented as log *k*_w_. A comparative evaluation was carried out between chromatographically-obtained lipophilicity parameters (represented as lipophilicity chromatographic index log *k*_w_ or isocratic chromatographic hydrophobicity index, $\varphi$_0_) and the computationally calculated log P values (miLogP, ALOGP, ACD/Labs and ALOGPs).

**Conclusion:**

It was found that a good correlation exists between the experimental and computed log P values. In the future, these results can give a deep insight about the anti-inflammatory and analgesic activity of the newly synthesized compounds.

**Electronic supplementary material:**

The online version of this article (10.1186/s13065-019-0607-6) contains supplementary material, which is available to authorized users.

## Introduction

Lipophilicity is a paramount descriptor which represents an essential part in design of new medications with required biological action [1]. It is also used in quantitative structure activity relationship (QSAR) investigations. The IUPAC (International Union of Pure and Applied Chemistry) defined it as “a physicochemical property which describes a partitioning equilibrium of solute molecules between water and an immiscible organic solvent” [2]. This property is one of the leading considerations as it has an important effect on pharmacodynamic and pharmacokinetic properties of drugs [3]. It suggests how the ADME (Absorption, Distribution, Metabolism, and Elimination) features of medications will proceed, in addition to its influence on their toxicological profile [4]. For its vital role on drug discovery and design process, the estimation of lipophilicity and how to regulate it has turned out to be routine procedure in drug development field [5]. Lipophilicity is also important in demonstrating the destiny of a compound in the environment, where it influences the bioavailability and bio-concentration in the food chain via sorption from water, and soil or dregs, which makes it an essential issue in danger evaluation and controlling of risky resources [6].

Lipophilicity is a fundamental molecular property which is defined as the logarithm of the octanol–water partition coefficient (log *P*_OW_), which is, in turn, expressed as the non-ionized drug spread ratio between the two phases of octanol and water [7]. log *P* measurements by different experimental protocols are illustrated in detail by the Organization for Economic Cooperation and Development (OECD) guidelines as test No. 107, which is Shake Flask method [8], and Test No. 123, which is the Slow Stirring method [9]. These traditional procedures are time-consuming, limited to extremely pure compound and need specific reagent to be performed. Hence, they have recently been replaced by more adaptable simpler methodologies which are chromatographic techniques (the OECD Test No. 117 [10]) that provide coherent results in the same log *P* range.

In the last few years, a great interest has been given for RP-HPLC as a tool for lipophilicity estimation and for characterization of pharmacological activity of molecules [11–13]. This is attributed to the close relationship between the retention performance of molecules in reversed phase chromatographic system and its lipophilicity [14]. The major advantages in using HPLC as a superior strategy are: smaller sample amount, high sensitivity to impurities, reproducibility and accuracy in addition to short determination time. The majority of the chromatographic systems used for measuring the lipophilicity rely on hydrophobic reversed-phase silica-based stationary phase (C_8_ or C_18_) [3].

In parallel to HPLC methods, computational methodologies have also been employed. Their wide use is based on considering the industrial requests as being simple, low cost, fast, and consistent approach to deliver information for fast screening of compounds under focus [5].

Celecoxib,4-[5-(4-methylphenyl)-3-(trifluoromethyl)pyrazol-1yl]benzenesulfonamide [15] is a selective inhibitor of cyclooxygenase-2 (COX-2) enzyme that is prescribed as a non-steroidal anti-inflammatory, analgesic, and antipyretic drug [16]. Being a selective cyclooxygenase-2 inhibitor, celecoxib seems to show fewer side effects than non-selective non-steroidal anti-inflammatory agents [17]. Unlike celecoxib, these non-selective agents act as inhibitors of both COX-1 and COX-2 enzymes which increases the possibility of inducing gastric ulcers, gastric bleeding and suppressed renal functions [18]. On the other hand, celecoxib has been alerted by FDA for its adverse side effects on cardiovascular system, and that represents the main motivation for discovering novel compounds with COX-2 inhibitory activity [19].

**Fig. 1:**
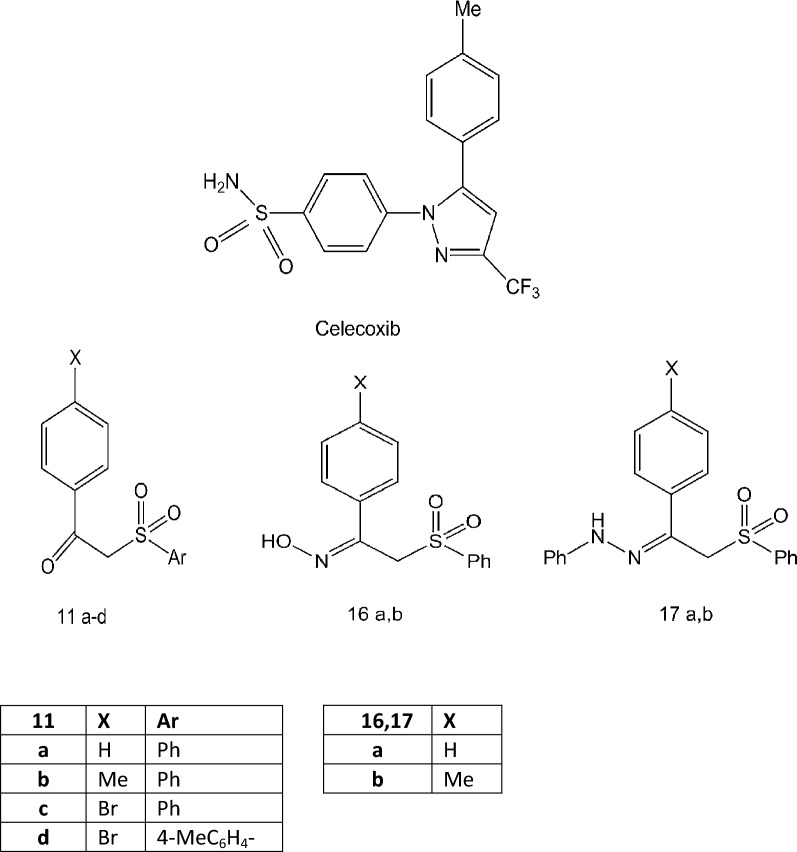
Chemical structures of the studied compounds

The principal aim of the present investigation is to develop an appropriate and proficient strategy for estimating the lipophilicity of a series of recently discovered *N*-benzenesulfonamide-1*H*-pyrazoles, oximes and hydrazones as celecoxib analogues [19] by RP-HPLC method and comparing these results with those obtained from computational programs. All the compounds in this series are weakly basic in nature (pK_a_ of celecoxib is 11.1) [15]. The investigated compounds are named 11a, 11b, 11c, 11d, 16a, 16b, 17a, and 17b in addition to celecoxib. The chemical structures of compounds under investigation are depicted in Fig. 1. Additional file 1 included H-NMR for the newly synthesized compounds.

## Experimental

### Reagents and chemicals

Celecoxib of purity 99.44% was kindly provided by Amoun Pharmaceutical Co. (Cairo, Egypt). The synthesis and characterization of the studied compounds (celecoxib analogues) were previously reported [16]. HPLC grade methanol was purchased from Sigma-Aldrich (Seelze, Germany). Double-distilled water was utilized throughout the study and was filtered through a Millipore membrane filter of bore size 0.45 μm (Additional file 1).

### Instrumentation and chromatographic conditions

For chromatographic analysis, stock solutions (200 μg/mL) of each of the investigated samples were prepared in methanol. HPLC measurements were performed utilizing Shimadzu Prominence HPLC system (Shimadzu Corporation, Japan) consisting of LC-20 AD pump, DGU-20 A5 degasser, CBM-20A interface, and SPD-20A UV–Vis detector with a 20 µL injection loop. The column utilized for studying the chromatographic behavior of the analytes was the most frequently used for lipophilicity investigations: Waters symmetry^®^ C_18_ column [250 mm × 4.6 mm (i.d.), 5-μm particle size]. The flow rate was 1.2 mL/min at room temperature. The retention behaviour of the analytes was investigated as a function of mobile phase composition ranging from 60–80% methanol and 40–20% water. The HPLC analyses were carried out at room temperature under isocratic conditions. Membrane filters (0.45 μm) from Sartorius (Goettingen, Germany) were used for filtration of samples. The absorbance of the analytes during a chromatographic run was performed at 200 nm. Each experiment was run in duplicate. Chromatographic data were collected using LabSolutions CS software. H^1^NMR spectra were recorded using Bruker NMR *spectrometer* (Bruker GmbH, Germany),

### Computational programs

Computational methods were performed using CS ChemDraw Ultra software (Cambridge Soft Corporation, Cambridge, MA, USA) running under Windows 7 operating system.

Computational lipophilicity (clog P) was calculated by the Advanced Chemistry Development ACD/Labs online service (https://ilab.acdlabs.com/iLab2/), Molinspiration online service (miLOGP, performed online at http://www.molinspiration.com/) and ALOGPS 2.1 online service (ALOGPS, performed online at http://www.vcclab.org/lab/alogps/).

## Results and discussion

### Theoretical aspects

It was shown from previous reports that the chromatographic systems using methanol: water as mobile phases were better for log *k* modeling than those containing acetonitrile as an organic modifier [4]. Moreover, it was found that the most preferred packing material for reversed-phase columns in lipophilicity assessment is C_18_ silanized silica gel. Chromatographic techniques including HPLC are based on the determination of the retention parameters [4]. The indices of lipophilicity measured by HPLC are obtained from the logarithm of the retention factor (log *k*), which is calculated by the following equation:

1 $$\log \;{k} = \log \frac{{{\text{t}}_{\text{r}} - {\text{t}}_{0} }}{{{\text{t}}_{0} }}$$

where; *t*_*R*_ is the retention time of the solute and *t*_*0*_ is the retention time of unretained species [20].

log *k* is proved experimentally to be in a linear relationship to the volume fraction of the organic solvent in the mobile phase ($\varphi$) according to the following linear regression equation [21]:

2 $$\log \;k = \log \;{\text{k}}_{\text{w}} - {\text{S}}{\varphi }$$

where; log k_w_ is the intercept of the linear regression curve which reflects the lipophilicity chromatographic index and denotes the factor of retention for pure water as eluent, and the slope (S) is extensively related to the strength of solvent or with specific hydrophobic surface area of solutes, and $\varphi$ is the portion of organic modifier volume.

Estimating log k_w_ directly is often very difficult, and is nearly non-feasible, because it can produce elongated retention time and also extreme peak broadening. Hence, estimating *k* with various proportions of water-organic solvent combination as mobile phases is favored, then extrapolating the correlation between log *k* and percentage of organic modifier specifies the value of log *k* upon utilizing pure water as mobile phase [22]. log *k* was estimated for each of the new celecoxib analogues at different proportions of methanol. Consequently, to obtain log k_w,_ an extrapolation to 100% of pure water was carried out. Then, linear regression analysis was performed [5]. Linear extrapolation is favored using definite ratios of organic modifier consistent with the lipophilicity level of the solutes [23].

The retention data were expressed as logarithm of the retention factor (log *k*) as shown in Table 1. It is clear that the lower the volume fraction of the organic modifier, the lower the elution power of the mobile phase and the higher the value of log *k*. The highest log *k* values were obtained when 60% v/v methanol was utilized.

**Table 1:** Logarithm of the retention factor (log *k*) on C_18_ column using methanol–water system (60–80% are percentages of methanol in the mobile phase)

Compound	Log *k*				
	60%	65%	70%	75%	80%
**11a**	0.088	0.041	− 0.206	− 0.412	− 0.695
**11b**	0.317	0.493	− 0.016	− 0.235	− 0.607
**11c**	0.635	0.501	0.123	− 0.036	− 0.145
**11d**	^a^	0.901	0.364	0.123	− 0.041
**16a**	0.130	0.053	− 0.220	− 0.424	− 0.385
**16b**	0.481	0.280	0.150	− 0.233	− 0.198
**17a**	0.950	0.824	0.740	− 0.12	− 0.051
**17b**	0.847	0.743	0.611	− 0.221	− 0.410
Celecoxib	^a^	0.950	0.83	0.530	0.235

^a^Compound retained on the stationary phase

**Fig. 2:**
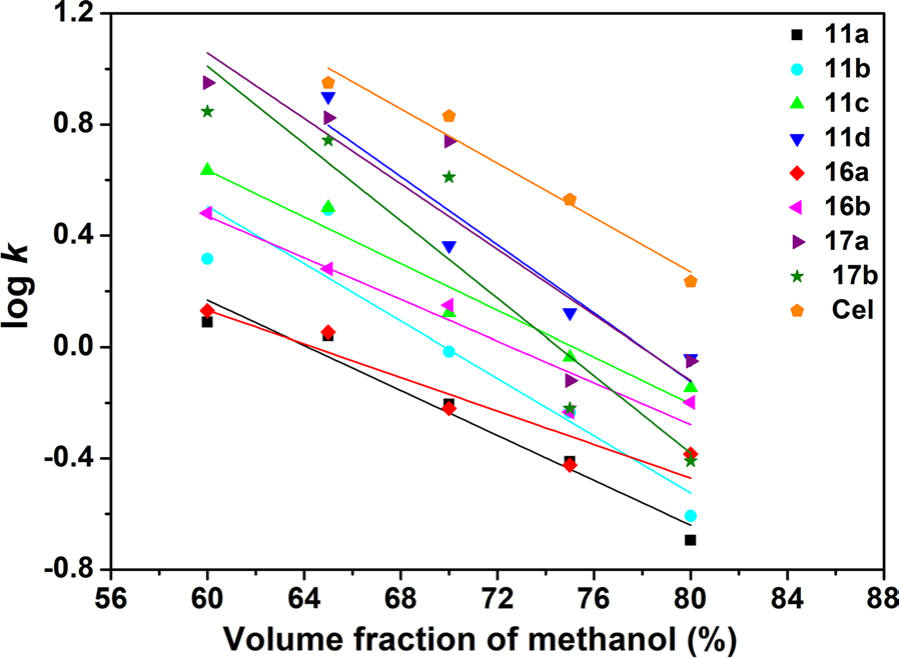
Linear fitting parameters of the relationship between log *k* and volume faction of methanol for nine of the studied compounds

The chromatographic data obtained for the nine investigated compounds together with the results of linear regression analysis are listed in Table 2 and represented in Fig. 2. For all examined drugs, high values of correlation coefficients were achieved (*r* > 0.9098), with small values of standard error of estimate, which proves the high significance of Eq. (2) for determination of lipophilicity. S is related to the specific hydrophobic surface area of solutes and it relies on the solute and the chromatographic system. A good correlation exists between log k_w_ and slope with *r* ≈ − 0.9716 which suggests a similar chromatographic retention mechanism for this homologous series of studied compounds. This relationship can be represented by the following equation:

3 $$\log \;{\text{k}}_{\text{w}} = - 85.342\left( { \pm 7.852} \right){\text{S}} - 0.536\left( { \pm 0.395} \right)$$

(*r* = 0.9716, R^2^ = 0.9440, F = 118.12, p value < 0.001, S_yx_ = 0.2822, n = 9), R^2^ is the determination coefficient, F is the value of test F- Snedecora, S_yx_ is standard error of estimate.

**Table 2:** Linear regression parameters between the logarithm of the retention factor (log *k*) and methanol volume fraction (**φ**)

Compound	log k = log k_w_ − S$\varphi$									
	Log k_w_	SD_a_	S	SD_b_	*r*	R^2^	S_yx_	F	$\varphi$_0_	Range
**11a**	2.593	± 0.331	− 0.040	± 0.005	0.9804	0.9610	0.074	74.10	64.83	60–80
**11b**	3.597	± 0.826	− 0.052	± 0.012	0.9301	0.8650	0.186	19.25	69.81	60–80
**11c**	3.151	± 0.359	− 0.042	± 0.005	0.9785	0.9570	0.081	67.59	75.14	60–80
**11d**	4.784	± 0.887	− 0.061	± 0.012	0.9627	0.9270	0.136	25.30	77.99	65–80
**16a**	1.941	± 0.413	− 0.030	± 0.006	0.9475	0.8980	0.092	26.34	64.39	60–80
**16b**	2.715	± 0.442	− 0.037	± 0.006	0.9602	0.9220	0.099	35.47	72.57	60–80
**17a**	4.590	± 1.091	− 0.059	± 0.015	0.9098	0.8280	0.245	14.41	77.97	60–80
**17b**	5.182	± 1.017	− 0.069	± 0.014	0.9409	0.8850	0.229	23.14	74.52	60–80
Celecoxib	4.180	± 0.445	− 0.048	± 0.006	0.9847	0.9696	0.068	63.81	85.51	65–80

SD_a_, standard deviation of intercept; SD_b_, standard deviation of slope, r, correlation coefficient, R^2^, determination coefficient, S_yx_, standard error of estimate (SEE); F, value of test F Snedecora at P value < 0.05; $\varphi$_0_, the hydrophobicity index

Another substitute to the index of lipophilicity is the chromatographic index of hydrophobicity $\varphi$_0_ which was first given by Valkò et al. [24]. It is calculated simply by the equation: $\varphi$_0_ = − log k_w_/S. $\upvarphi_0$ denotes the volume fraction of the organic modifier in the mobile phase at which equal partitioning of the solute between the mobile and stationary phases is obtained, that is, k = 1, log *k *= 0 [22]. The value of $\varphi$_0_ is specific for each compound; it relies solely on the pH, organic modifier and temperature. It does not rely on the column type and length, flow rate and the composition of the mobile phase with which the retention determination are made. Moreover, $\varphi$_0_ can be accurately estimated as it possesses a solid physical meaning, that is, the concentration of the organic modifier in the mobile phase where the retention time is accurately double the dead time. This is considered a distinguishable advantage over extrapolating of log *k* values to pure water mobile phase [24]. $\varphi$_0_ values calculated for the studied compounds are listed in Table 2.

### Lipophilicity study

Eight compounds of the N-benzenesulfonamide-1H-pyrazoles, oximes and hydrazones series were investigated in this study as celecoxib analogues. They possess similar structural features with different substituents that significantly impact their physicochemical, pharmacokinetic, and pharmacodynamic properties. RP-HPLC was utilized to estimate their lipophilicity (conveyed as log k_w_ or $\varphi$_0_). In this study, C_18_ column was utilized as the stationary phase which is consisting of silica gel amended by a lipophilic C_18_ hydrocarbon chain while the mobile phase was more polar than the stationary phase. It is well-known that polar molecules have lower retention times and hence lower retention factors than non-polar molecules which are strongly attracted to the stationary phase hydrocarbon groups as explained by the Van der Waals forces [25].

For the first group of the studied compounds (aryl sulfones), in relation to the retention data, it can be observed that 11a is more polar than 11b. The methyl group substituent in 11b reduces its polarity. It is also noted that 11c is more polar than 11d due to the methyl substituent in the aryl moiety of the sulfone group in 11d. These observations coincide very well with the data obtained (Tables 1 and 2). Similarly, in the second and third groups of the studied compounds (oximes and hydrazones, respectively), due to the methyl substituent, compound 16a is more polar than 16b and compound 17a is more polar than compound 17b.

The linear regression parameters between log *k* values and methanol volume fraction are abridged in Table 2.

### Correlation between log K_w_ and φ_0_ and computed log P values

The following step in this work is to compare chromatographically-obtained lipophilicity parameters; log k_w_ or $\varphi$_0_ of the studied compounds with clog P calculated using different computational methods (that utilize different theoretical approaches, principles of which were described by Mornar et al. [26]) such as ACD/Labs, miLOGP, ALOGPS, and APLOGP. A very good correlation exists between log k_w_ and computed log P values (r in all cases ≥ 0.8283) as represented in Table 3, which confirms the reliability of HPLC technique as a tool for lipophilicity estimation. Although a fair correlation exists between $\varphi$_0_ and miLogP, a good correlation still presents between $\varphi$_0_ and the other computed log P values (Table 3), which is a further proof for the validity of this approach for determination of lipophilicity. All the investigated compounds purity was confirmed by the H^1^NMR spectrometer (Table 4).

**Table 3:** Correlation between experimental log k_w_ values or $\varphi$_0_ values and computed log P values (calculated using different techniques)

Compound	log k_w_	$\varphi$_0_	miLogP^a^	ALOGPS^b^	ACD/Labs^c^	APLOGP^c^
**11a**	2.59	64.83	1.96	2.20	2.28	2.07
**11b**	3.60	69.81	2.40	2.60	2.74	2.57
**11c**	3.15	75.14	2.76	3.05	3.04	2.92
**11d**	4.78	77.99	3.21	3.38	3.5	3.33
**16a**	1.94	64.39	2.02	1.86	2.42	2.62
**16b**	2.72	72.57	2.47	1.86	2.88	2.86
**17a**	4.59	77.97	5.33	4.37	4.12	3.83
**17b**	5.18	74.52	5.78	4.60	4.58	4.09
Celecoxib	4.18	85.51	3.61	3.99	4.61	3.92
r between log k_w_ values and computed log P values^d^			0.8397	0.9107	0.8501	0.8283
r between $\varphi$_0_ values and computed log P values^d^			0.5548	0.7263	0.8414	0.8143

Correlation between experimental log k_w_ values or $\varphi$_0_ values and computed log P values (calculated using different techniques)

^a^Calculated using molinspiration online service (http://www.molinspiration.com/)

^b^Calculated using ALOGPS 2.1 online service (http://www.vcclab.org/lab/alogps/)

^c^Calculated using ACD/Labs online service (https://ilab.acdlabs.com/iLab2/)

^d^Correlation coefficient

**Table 4:** Purity of the studied compounds

Compound number	Purity^a^
**11 a**	97.03
**11 b**	98.25
**11 c**	94.27
**11 d**	96.84
**16 a**	99.50
**16 b**	98.10
**17 a**	98.80
**17 b**	99.70

^a^The purity of the target compounds was confirmed by H^1^NMR using *Bruker* NMR *spectrometer* and DMSO as solvent. Charts describing compounds 16 a,b and 17 a,b were included to confirm the purity of the mentioned compounds. HPLC run was also performed to check and evaluate such purity as well

For further investigation of the differences among the computational and experimental lipophilicity, the lipophilicity values were arranged in a matrix of dimensions nine (compounds) × six (2 computational and four experimental lipophilicity (log k_w_ or $\varphi$_0_)), then principal component analysis PCA was performed on the whole matrix.

**Fig. 3:**
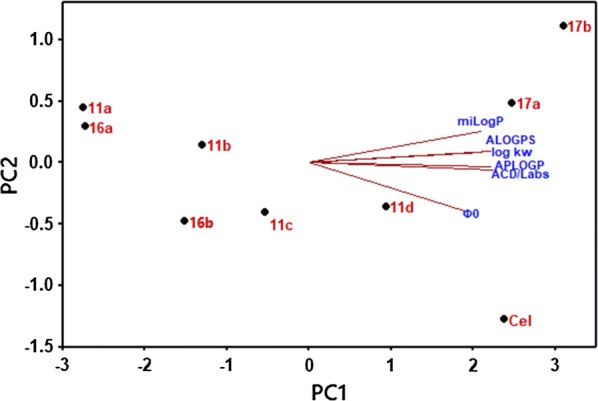
Scores and loadings of Principal Component Analysis of the lipophilicity matrix

The first principal component PC1 accounts for 86.7% of the data variation, whereas PC2 explains 8.1% of the data variation, respectively (94.8% total). As given in Fig. 3, the variation in the lipophilicity of the investigated analogues can be mainly explained by PC1, while PC2 explains differences among chromatographic and computational techniques, which strongly justify the utilization of chromatographic techniques for lipophilicity estimation as a promising substitute to experimental methods based on Shake-Flask method.

## Conclusion

In this work, determination of lipophilicity of celecoxib and its newly synthesized analogues was carried out by RP-HPLC using a C_18_ silanized silica gel stationary phase. This experimental study proved to be simple, reliable and does not need sophisticated or complicated methodologies which show the importance of this method for pharmaceutical applications. Lipophilicity is an essential physicochemical property that affects pharmacokinetics and pharmacodynamics as well as toxicity of drug molecules. Moreover, it is well known that this essential property is important for the in vivo distribution of organic compounds by influencing their solubility, oral absorption, cell uptake, blood–brain penetration and metabolism and to rationalizing a number of biological events as membrane penetration and permeability. Under suitable isocratic chromatographic conditions, extrapolated retention factors are in good correlation with the computationally calculated log P obtained using different software programs. Hence, RP-HPLC is an easily applicable technique for determination of lipophilicity and the chromatographic retention.

## Additional file

**Additional file 1.** H-NMR for the synthesized compounds.

## Data Availability

The data material are available whenever its needed from authors.

## Abbreviations

RP-HPLC: reversed-phase high performance liquid chromatography
log *P*_OW_: octanol–water partition coefficient
K: retention factor
$\varphi$_0_: the chromatographic index of hydrophobicity
