# Supplementary material for: Assessment of lipophilicity of newly synthesized celecoxib analogues using reversed-phase HPLC
Source: BMC Chem. 2019 Jul 9;13(1):84. doi: 10.1186/s13065-019-0607-6 (PMC6661952; doi:10.1186/s13065-019-0607-6)
Supplement: Supplementary file 1 — Additional file 1. H-NMR for the synthesized compounds. [file 13065_2019_607_MOESM1_ESM.pdf]

'-BBO DMSO D:\\ m

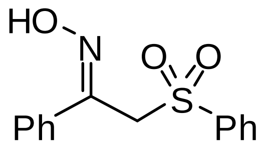

16a

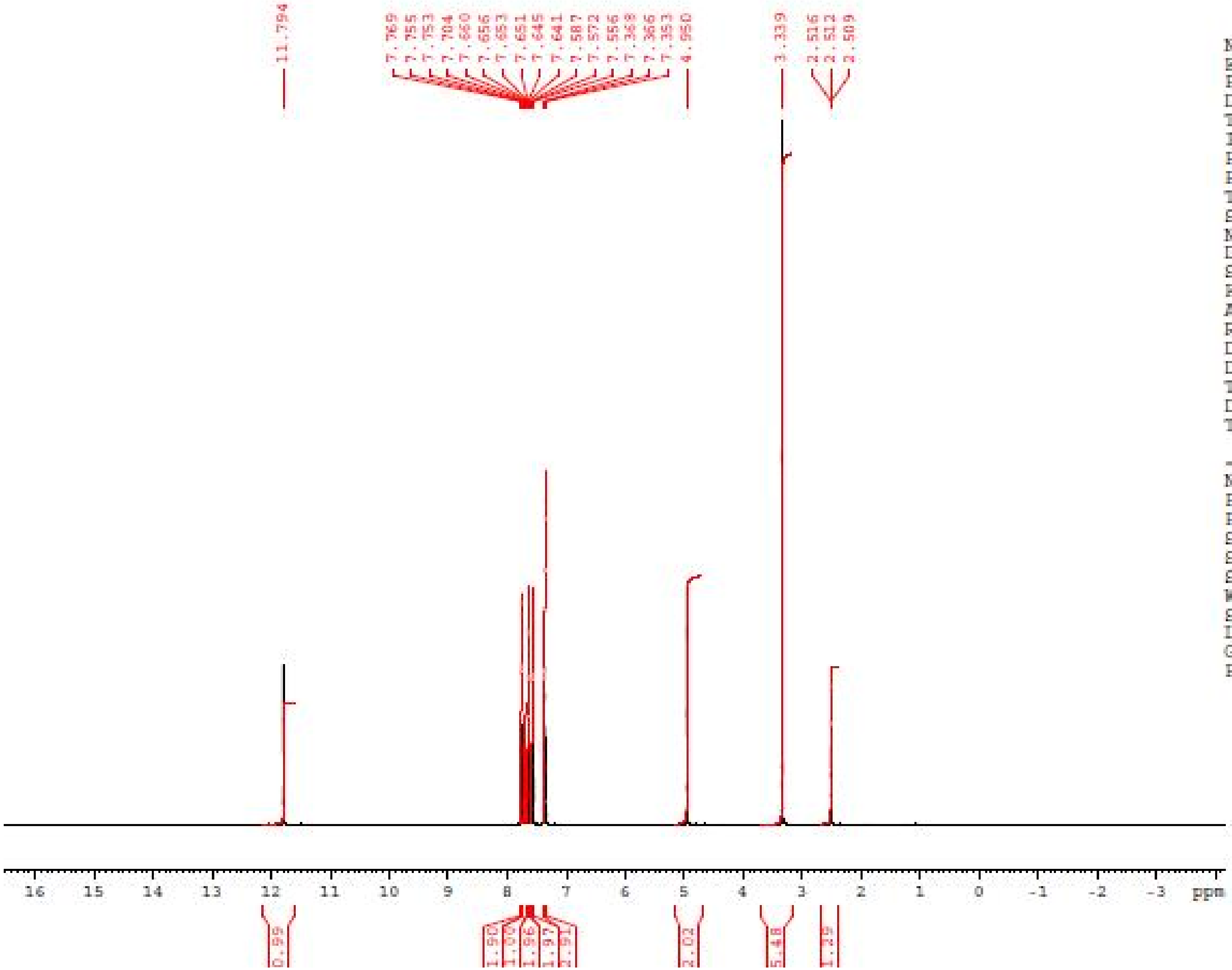

BRUKER

NAME	drhatem-4a
EXPNO	10
PROCNO	1
Date_	20130304
Time	22.22
INSTRUM	spect
PROBHD	5 mm PABBO BB-
PULPROG	zg30
TD	65536
SOLVENT	DMSO
NS	16
DS	2
SWH	10330.578 Hz
FIDRES	0.157632 Hz
AQ	3.1720407 sec
RG	161.3
DW	48.400 usec
DE	6.50 usec
TE	301.1 K
D1	1.00000000 sec
TD0	1
======== CHANNEL f1 ========	
NUC1	1H
P1	14.70 usec
PL1	-1.00 dB
SFO1	500.1330885 MHz
SI	32768
SF	500.1300000 MHz
WDW	EM
SSB	0
LB	0.30 Hz
GB	0
PC	1.00

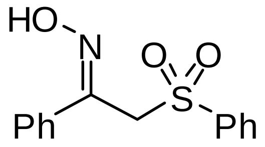
HO
N
O
O
S
Ph
Ph

16a

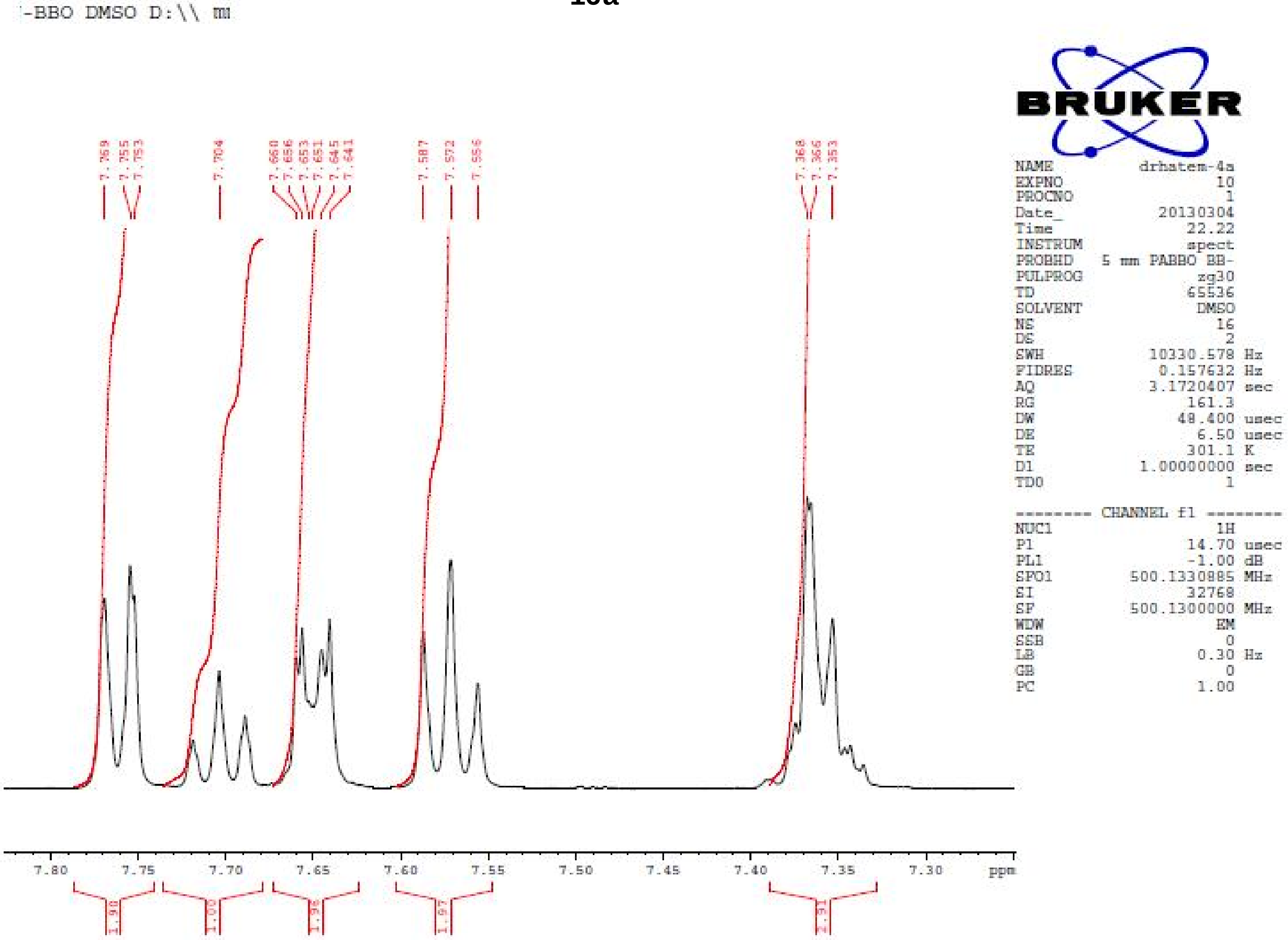
-BBO DMSO D:\\ mi
BRUKER
7.769
7.755
7.753
7.704
7.660
7.656
7.653
7.651
7.645
7.641
7.587
7.572
7.556
7.368
7.366
7.353
NAME drhatem-4a
EXPNO 10
PROCNO 1
Date_ 20130304
Time 22.22
INSTRUM spect
PROBHD 5 mm PABBO BB-
PULPROG zg30
TD 65536
SOLVENT DMSO
NS 16
DS 2
SWH 10330.578 Hz
FIDRES 0.157632 Hz
AQ 3.1720407 sec
RG 161.3
DW 48.400 usec
DE 6.50 usec
TE 301.1 K
D1 1.00000000 sec
TD0 1
======== CHANNEL f1 ========
NUC1 1H
P1 14.70 usec
PL1 -1.00 dB
SFO1 500.1330885 MHz
SI 32768
SF 500.1300000 MHz
WDW EM
SSB 0
LB 0.30 Hz
GB 0
PC 1.00
7.80
7.75
7.70
7.65
7.60
7.55
7.50
7.45
7.40
7.35
7.30
ppm
1.98
1.00
1.96
1.97
2.91

-BBO DMSO D:\\ m

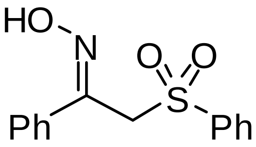

16a

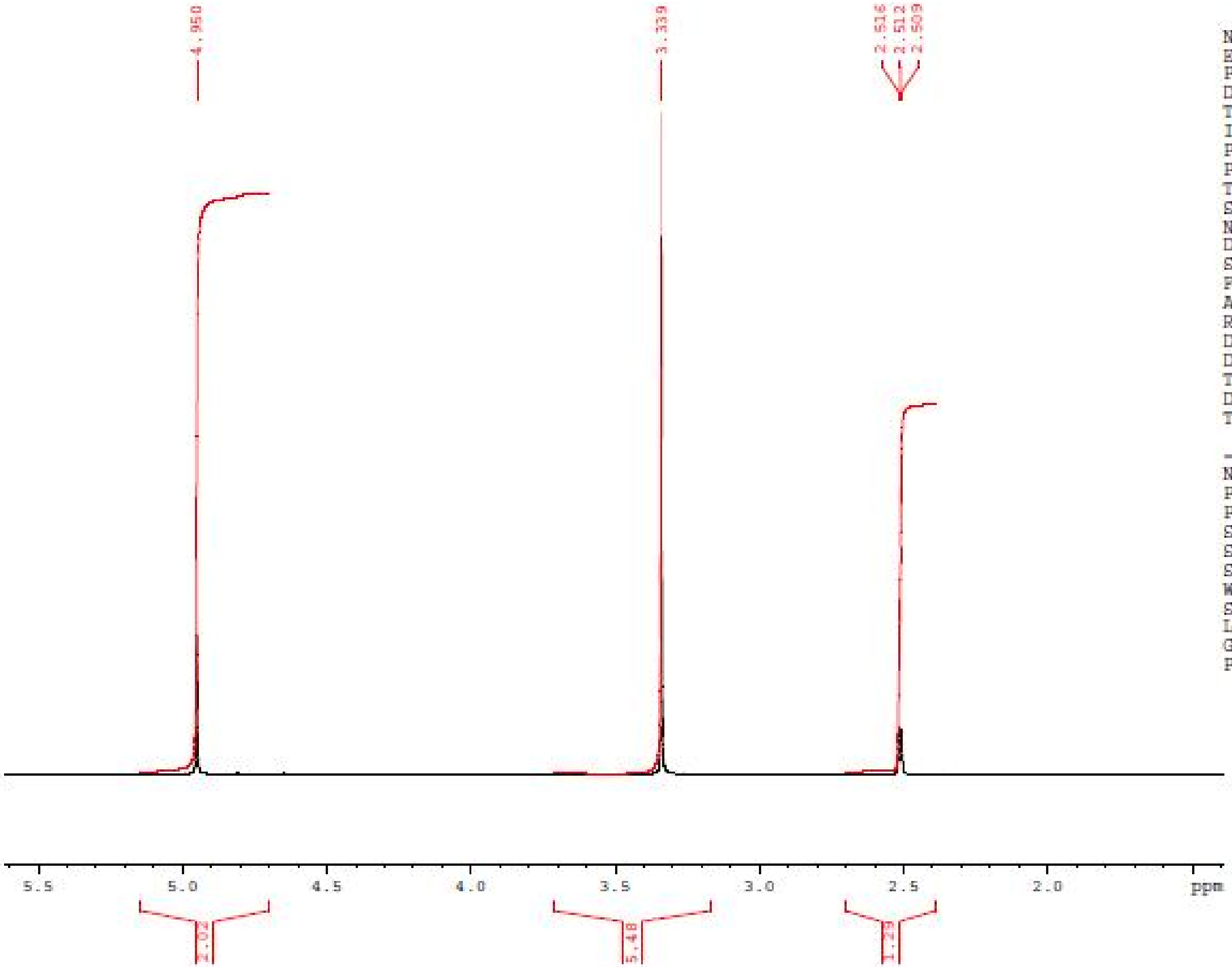

NAME	drhatem-4a
EXPNO	10
PROCNO	1
Date_	20130304
Time	22.22
INSTRUM	spect
PROBHD	5 mm PABBO BB-
PULPROG	zg30
TD	65536
SOLVENT	DMSO
NS	16
DS	2
SWH	10330.578 Hz
FIDRES	0.157632 Hz
AQ	3.1720407 sec
RG	161.3
DW	48.400 usec
DE	6.50 usec
TE	301.1 K
D1	1.00000000 sec
TD0	1

======== CHANNEL f1 ========

NUC1	1H
P1	14.70 usec
PL1	-1.00 dB
SFO1	500.1330885 MHz
SI	32768
SF	500.1300000 MHz
WDW	EM
SSB	0
LB	0.30 Hz
GB	0
PC	1.00

3BO DMSO D:\\ mmj

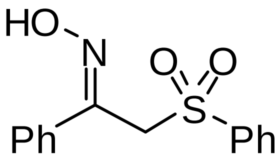

16a

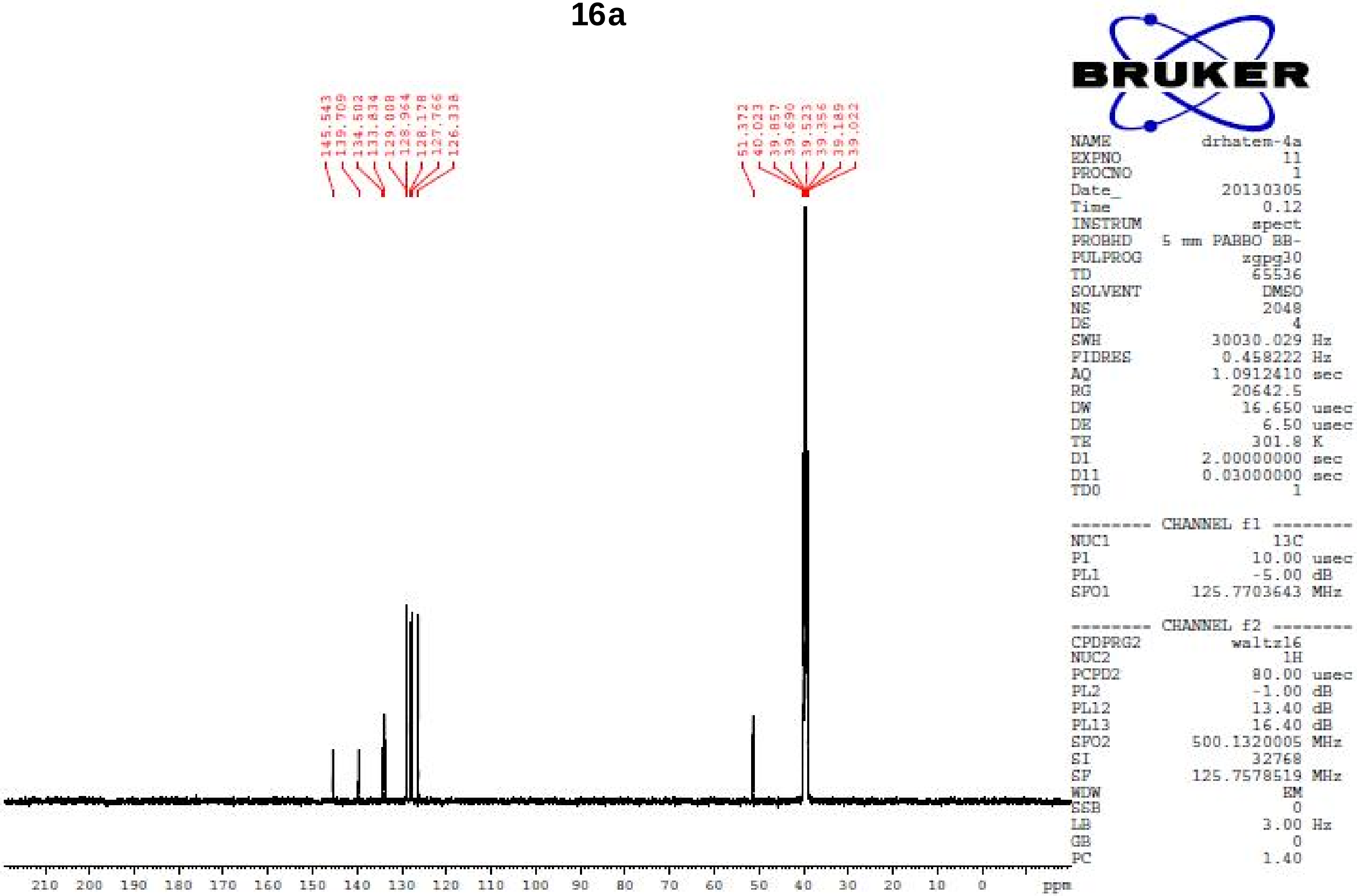

3BO DMSO D:\\ mmj

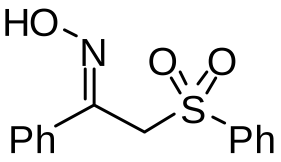

16a

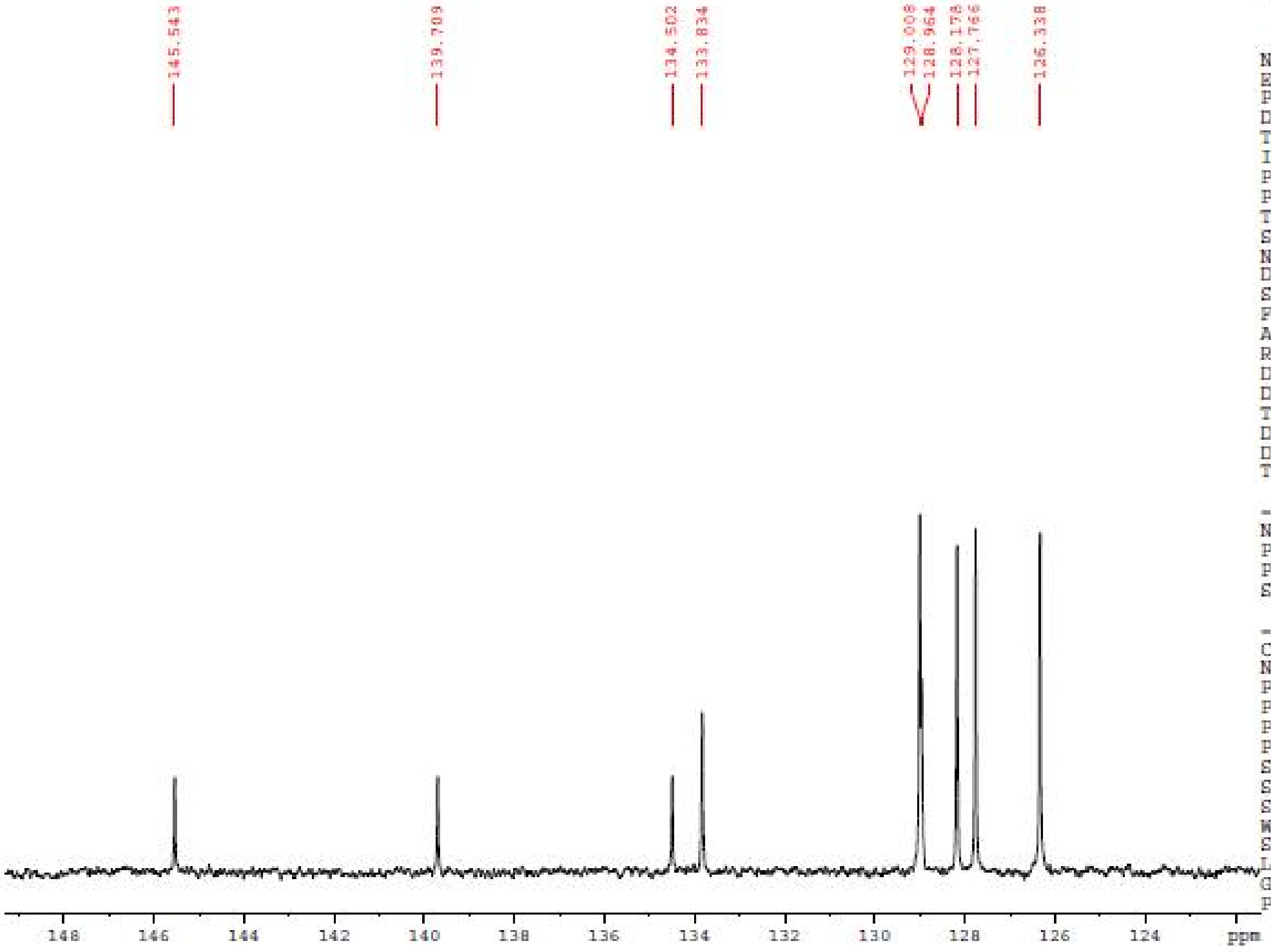

BRUKER

NAME	drhatem-4a
EXPNO	11
PROCNO	1
Date_	20130305
Time	0.12
INSTRUM	spect
PROBHD	5 mm PABBO BB-
PULPROG	zgpg30
TD	65536
SOLVENT	DMSO
NS	2048
DS	4
SWH	30030.029 Hz
FIDRES	0.458222 Hz
AQ	1.0912410 sec
RG	20642.5
DW	16.650 usec
DE	6.50 usec
TE	301.8 K
D1	2.00000000 sec
D11	0.03000000 sec
TD0	1

======== CHANNEL f1 ========

NUC1	13C
P1	10.00 usec
PL1	-5.00 dB
SFO1	125.7703643 MHz

======== CHANNEL f2 ========

CPDPRG2	waltz16
NUC2	1H
PCPD2	80.00 usec
PL2	-1.00 dB
PL12	13.40 dB
PL13	16.40 dB
SFO2	500.1320005 MHz
SI	32768
SF	125.7578519 MHz
WDW	EM
SSB	0
LB	3.00 Hz
GB	0
PC	1.40

-BBO DMSO D:\\ m

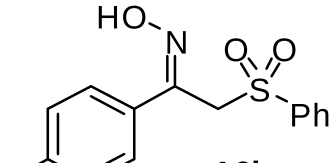

16b

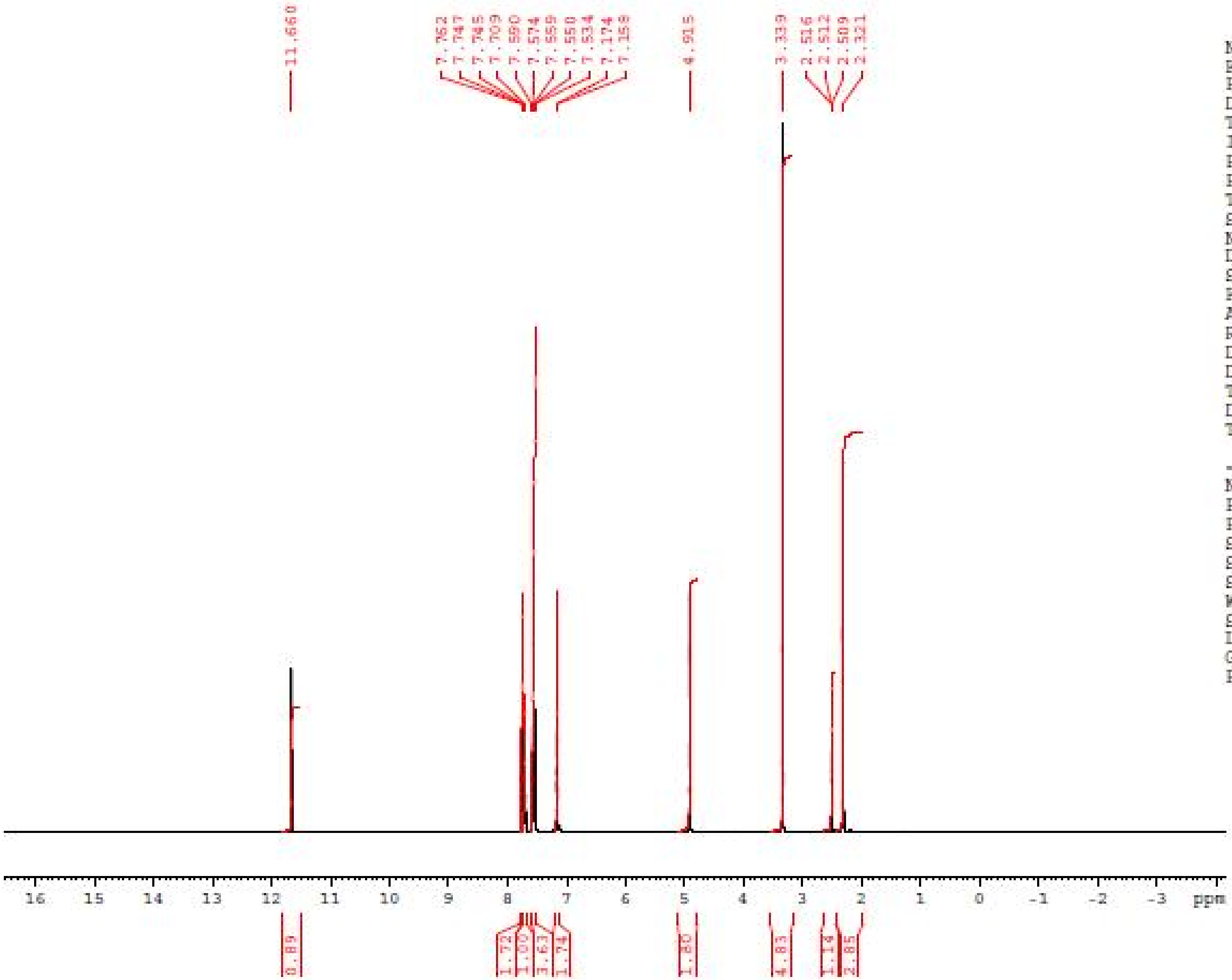

BRUKER

NAME	drhatem-4b
EXPNO	10
PROCNO	1
Date_	20130304
Time	20.28
INSTRUM	spect
PROBHD	5 mm PABBO BB-
PULPROG	zg30
TD	65536
SOLVENT	DMSO
NS	16
DS	2
SWH	10330.578 Hz
FIDRES	0.157632 Hz
AQ	3.1720407 sec
RG	161.3
DW	48.400 usec
DE	6.50 usec
TE	301.1 K
D1	1.00000000 sec
TD0	1

======== CHANNEL f1 ========

NUC1	1H
P1	14.70 usec
PL1	-1.00 dB
SFO1	500.1330885 MHz
SI	32768
SF	500.1300000 MHz
WDW	EM
SSB	0
LB	0.30 Hz
GB	0
PC	1.00

-BBO DMSO D:\\ mi

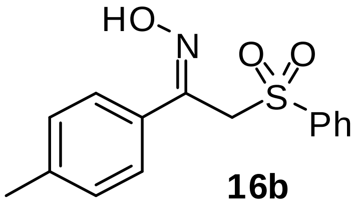

16b

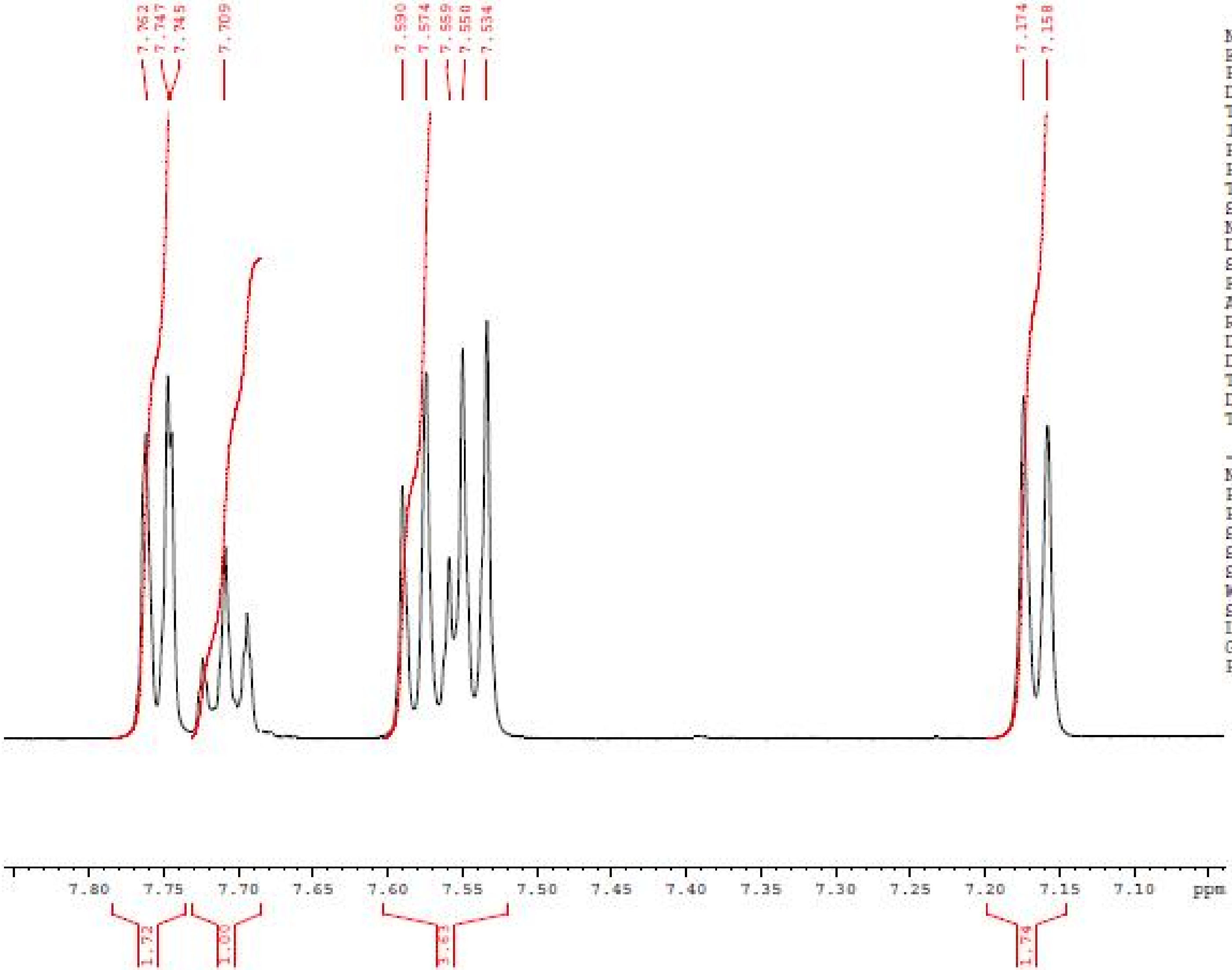

NAME	drhatem-4b
EXPNO	10
PROCNO	1
Date_	20130304
Time	20.28
INSTRUM	spect
PROBHD	5 mm PABBO BB-
PULPROG	zg30
TD	65536
SOLVENT	DMSO
NS	16
DS	2
SWH	10330.578 Hz
FIDRES	0.157632 Hz
AQ	3.1720407 sec
RG	161.3
DW	48.400 usec
DE	6.50 usec
TE	301.1 K
D1	1.00000000 sec
TD0	1

======== CHANNEL f1 ========

NUC1	1H
P1	14.70 usec
PL1	-1.00 dB
SFO1	500.1330885 MHz
SI	32768
SF	500.1300000 MHz
WDW	EM
SSB	0
LB	0.30 Hz
GB	0
PC	1.00

-BBO DMSO D:\\ mm

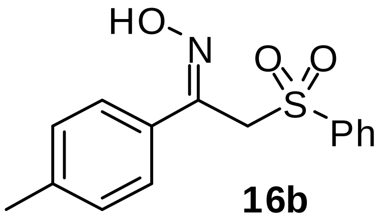

16b

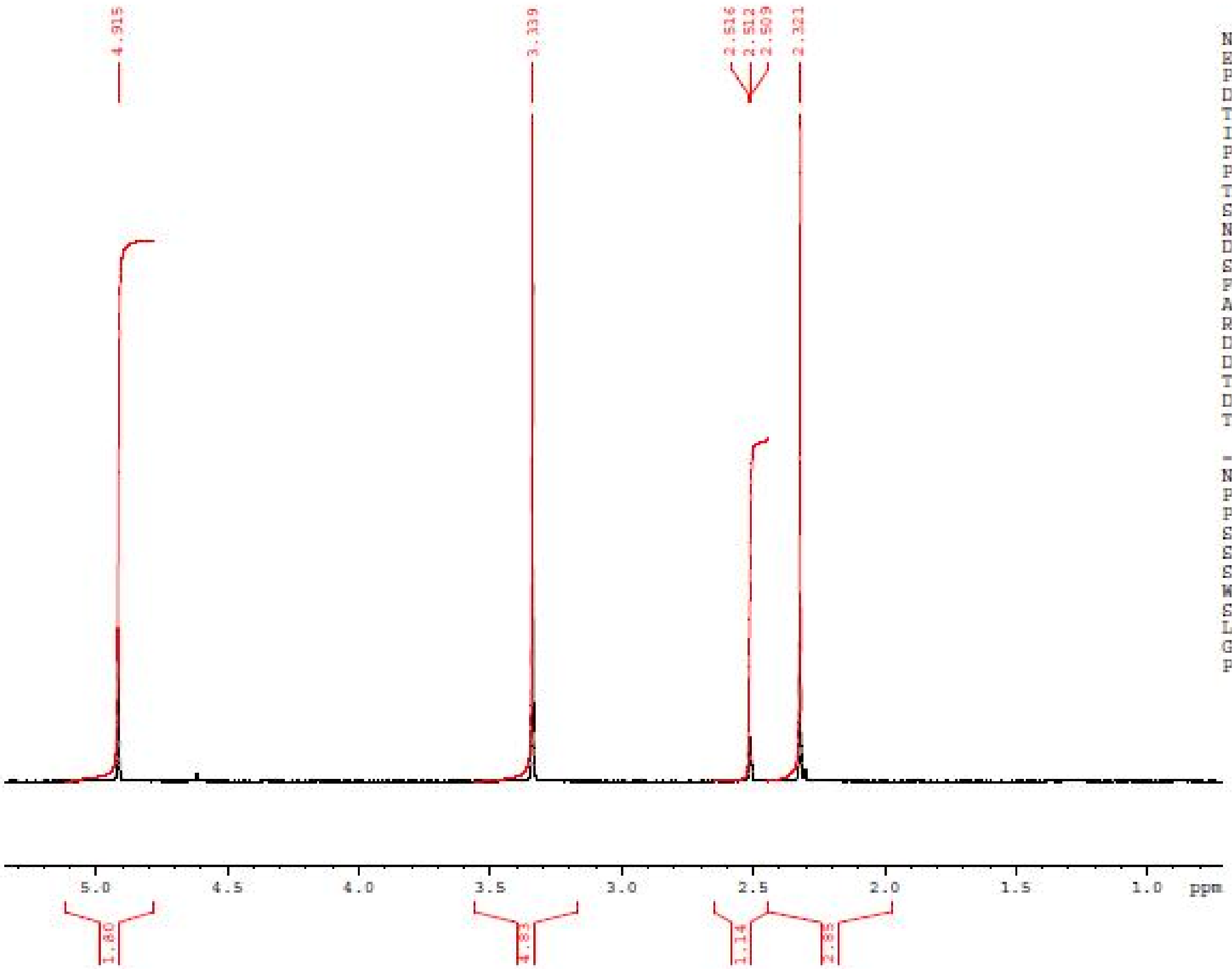

NAME	drhatem-4b
EXPNO	10
PROCNO	1
Date_	20130304
Time	20.28
INSTRUM	spect
PROBHD	5 mm PABBO BB-
PULPROG	zg30
TD	65536
SOLVENT	DMSO
NS	16
DS	2
SWH	10330.578 Hz
FIDRES	0.157632 Hz
AQ	3.1720407 sec
RG	161.3
DW	48.400 usec
DE	6.50 usec
TE	301.1 K
D1	1.00000000 sec
TD0	1
======== CHANNEL f1 ========	
NUC1	1H
P1	14.70 usec
PL1	-1.00 dB
SFO1	500.1330885 MHz
SI	32768
SF	500.1300000 MHz
WDW	EM
SSB	0
LB	0.30 Hz
GB	0
PC	1.00

3BO DMSO D:\\ mmj

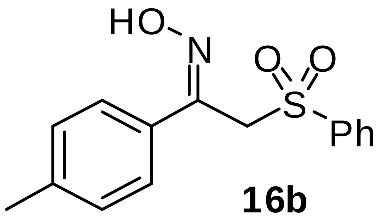

16b

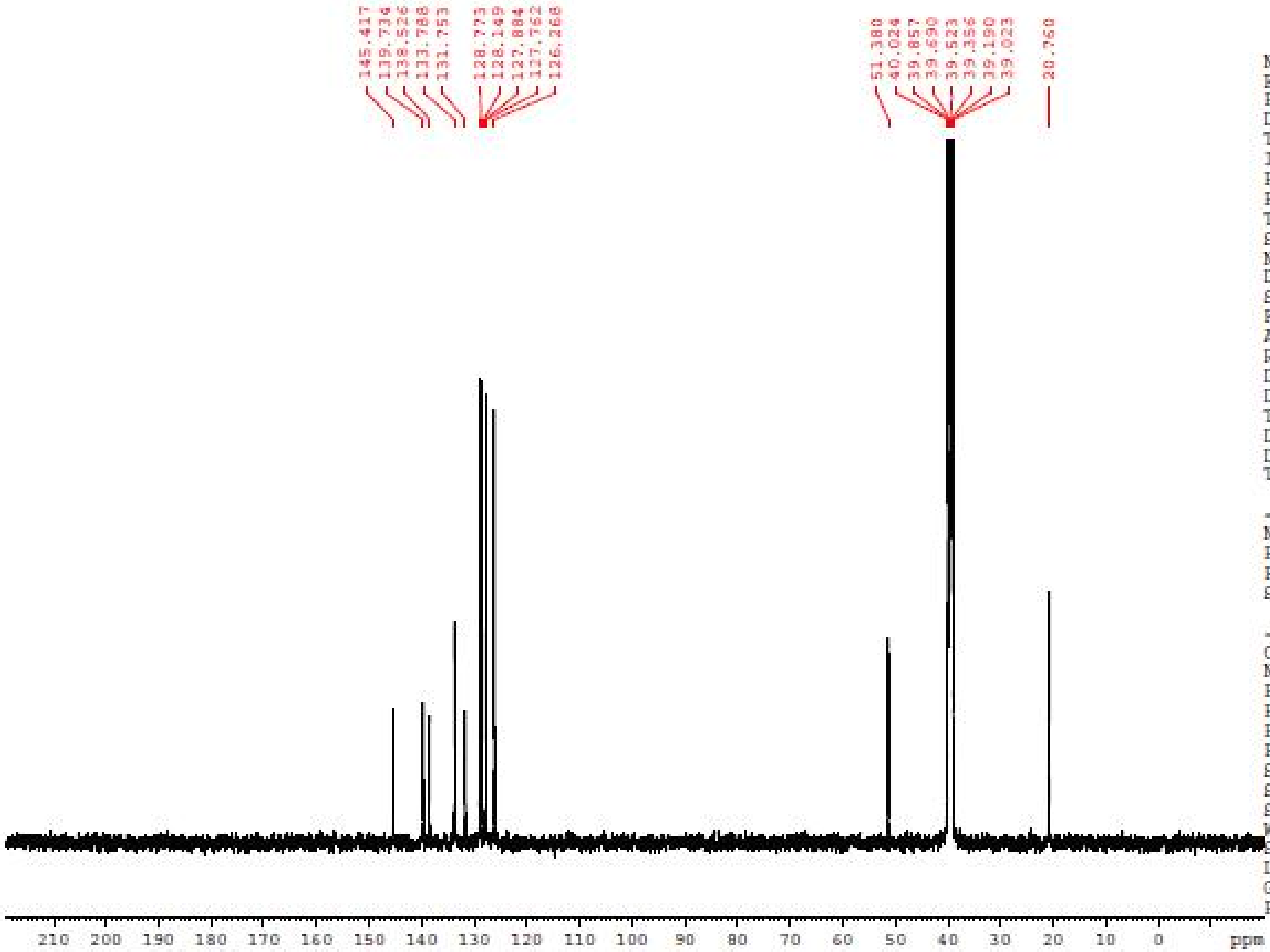

NAME	drhatem-4b
EXPNO	11
PROCNO	1
Date_	20130304
Time	22.18
INSTRUM	spect
PROBHD	5 mm PABBO BB-
PULPROG	zgpg30
TD	65536
SOLVENT	DMSO
NS	2048
DS	4
SWH	30030.029 Hz
FIDRES	0.458222 Hz
AQ	1.0912410 sec
RG	14596.5
DW	16.650 usec
DE	6.50 usec
TE	301.9 K
D1	2.00000000 sec
D11	0.03000000 sec
TD0	1
======== CHANNEL f1 ========	
NUC1	13C
P1	10.00 usec
PL1	-5.00 dB
SFO1	125.7703643 MHz
======== CHANNEL f2 ========	
CPDPRG2	waltz16
NUC2	1H
PCPD2	80.00 usec
PL2	-1.00 dB
PL12	13.40 dB
PL13	16.40 dB
SFO2	500.1320005 MHz
SI	32768
SF	125.7578519 MHz
WDW	EM
SSB	0
LB	3.00 Hz
GB	0
PC	1.40

3BO DMSO D:\\ mmj

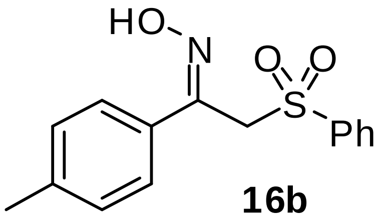

16b

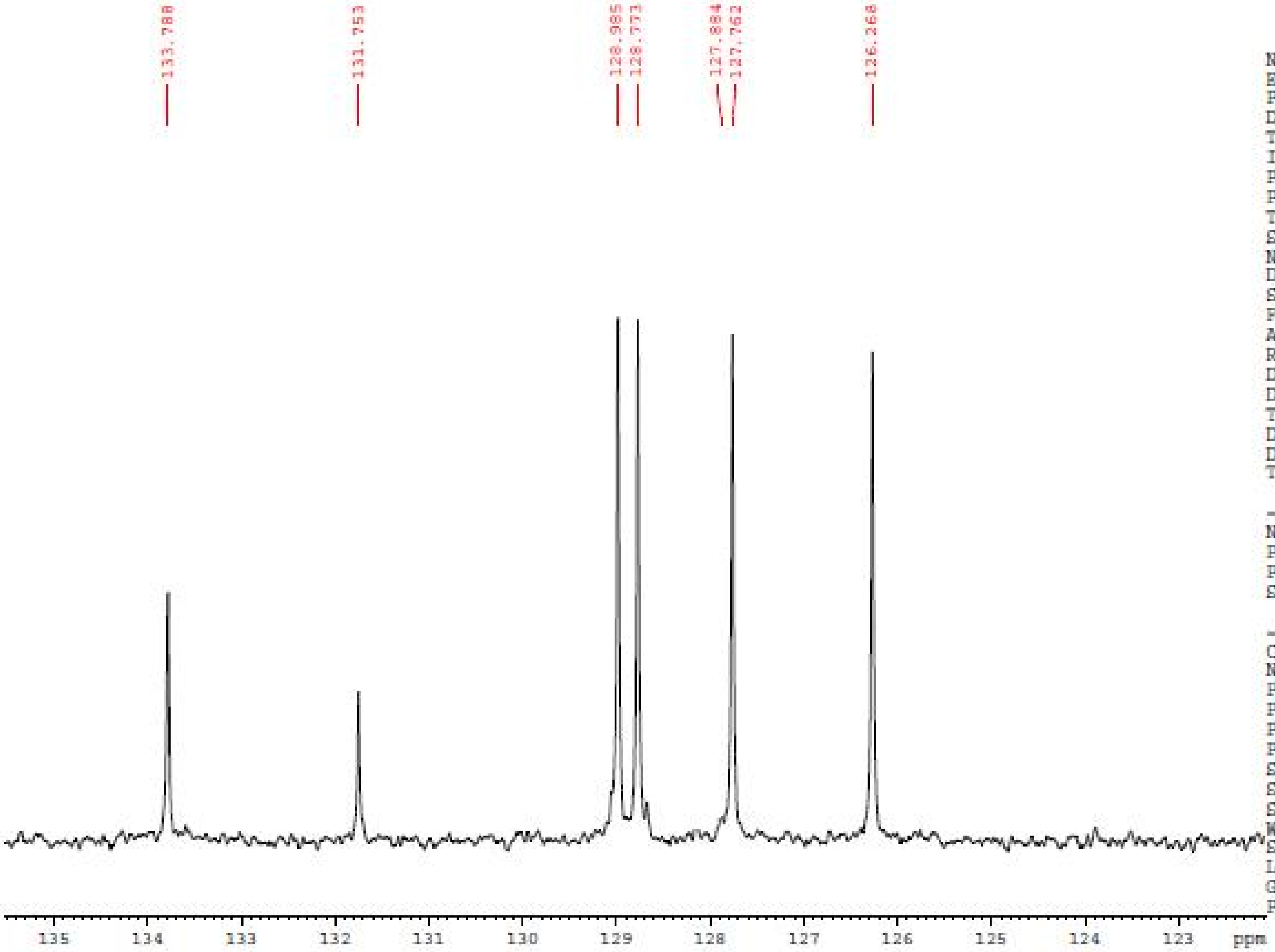

NAME	drhatem-4b
EXPNO	11
PROCNO	1
Date_	20130304
Time	22.18
INSTRUM	spect
PROBHD	5 mm PABBO BB-
PULPROG	zgpg30
TD	65536
SOLVENT	DMSO
NS	2048
DS	4
SWH	30030.029 Hz
FIDRES	0.458222 Hz
AQ	1.0912410 sec
RG	14596.5
DW	16.650 usec
DE	6.50 usec
TE	301.9 K
D1	2.00000000 sec
D11	0.03000000 sec
TD0	1
======== CHANNEL f1 ========	
NUC1	13C
P1	10.00 usec
PL1	-5.00 dB
SFO1	125.7703643 MHz
======== CHANNEL f2 ========	
CPDPRG2	waltz16
NUC2	1H
PCPD2	80.00 usec
PL2	-1.00 dB
PL12	13.40 dB
PL13	16.40 dB
SFO2	500.1320005 MHz
SI	32768
SF	125.7578519 MHz
WDW	EM
SSB	0
LB	3.00 Hz
GB	0
PC	1.40

-BBO DMSO D:\\ m

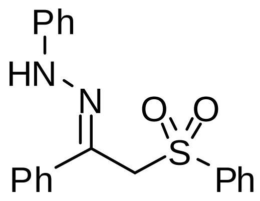

17a

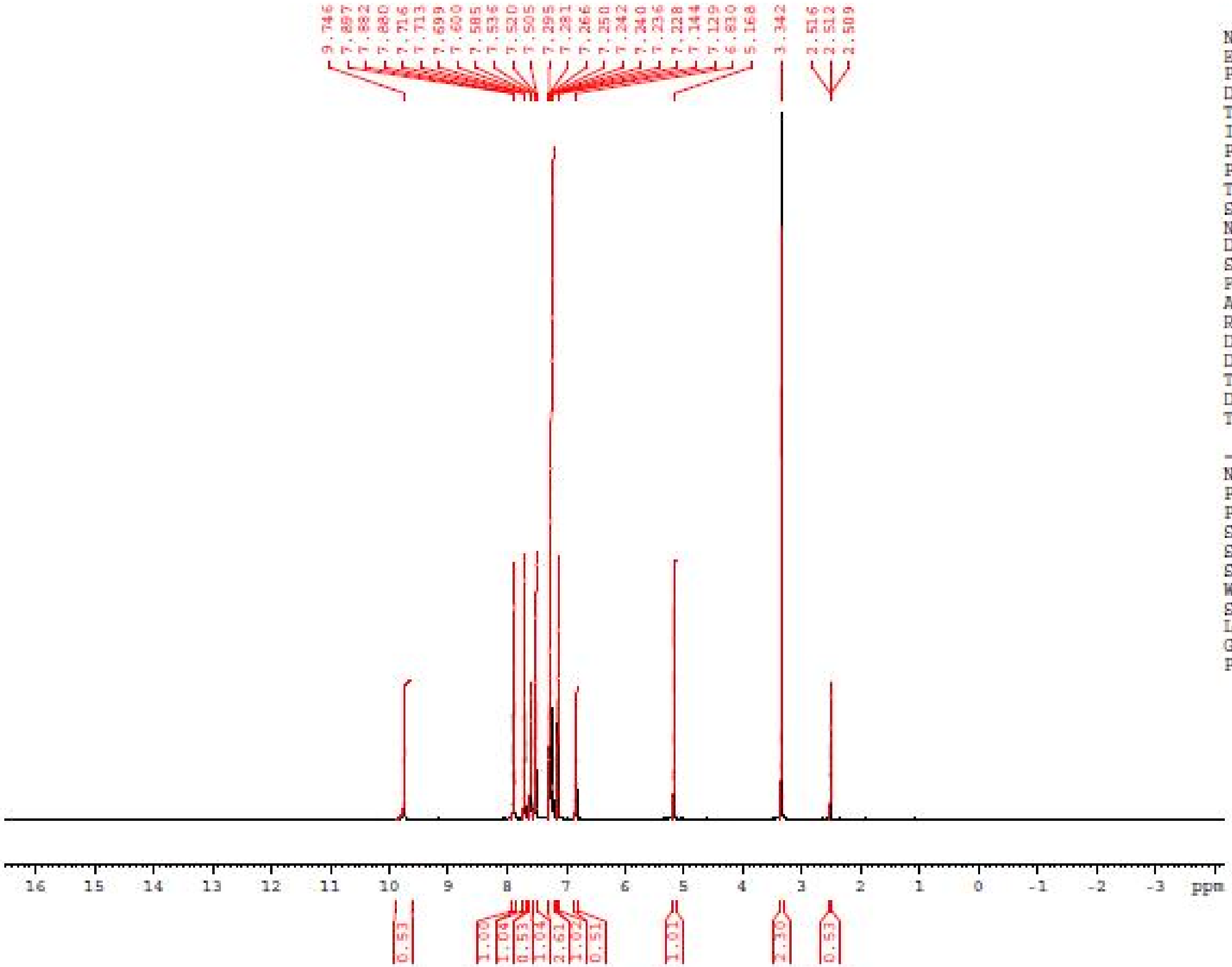

BRUKER

NAME	drhatem-5a
EXPNO	10
PROCNO	1
Date_	20130304
Time	16.39
INSTRUM	spect
PROBHD	5 mm PABBO BB-
PULPROG	zg30
TD	65536
SOLVENT	DMSO
NS	16
DS	2
SWH	10330.578 Hz
FIDRES	0.157632 Hz
AQ	3.1720407 sec
RG	128
DW	48.400 usec
DE	6.50 usec
TE	301.1 K
D1	1.00000000 sec
TD0	1

======== CHANNEL f1 ========

NUC1	1H
P1	14.70 usec
PL1	-1.00 dB
SFO1	500.1330885 MHz
SI	32768
SF	500.1300000 MHz
WDW	EM
SSB	0
LB	0.30 Hz
GB	0
PC	1.00

-BBO DMSO D:\\ m

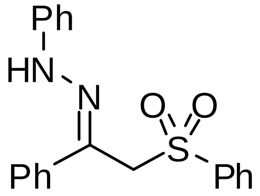

17a

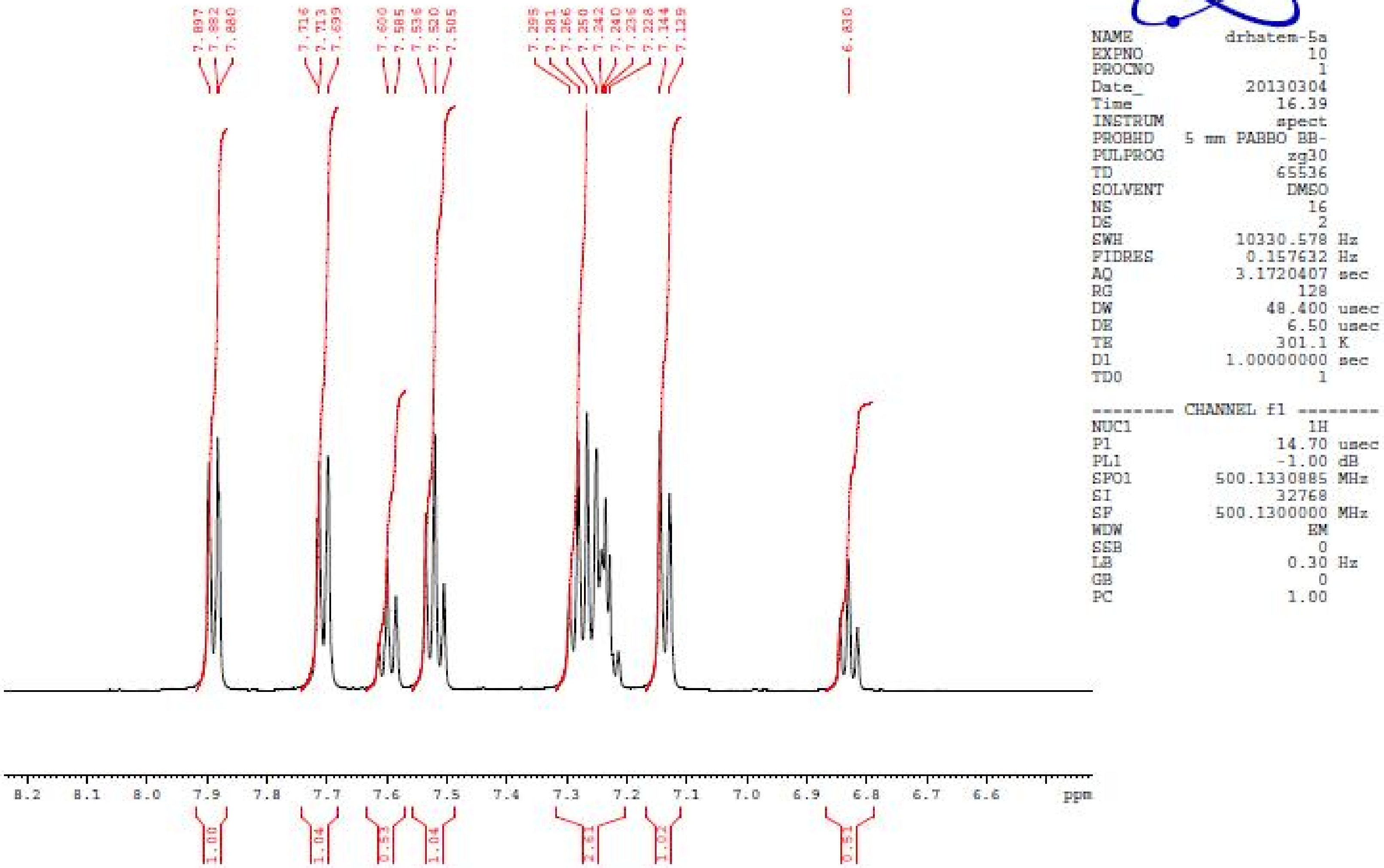

-BBO DMSO D:\\ m

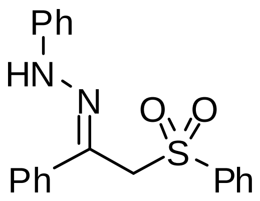

17a

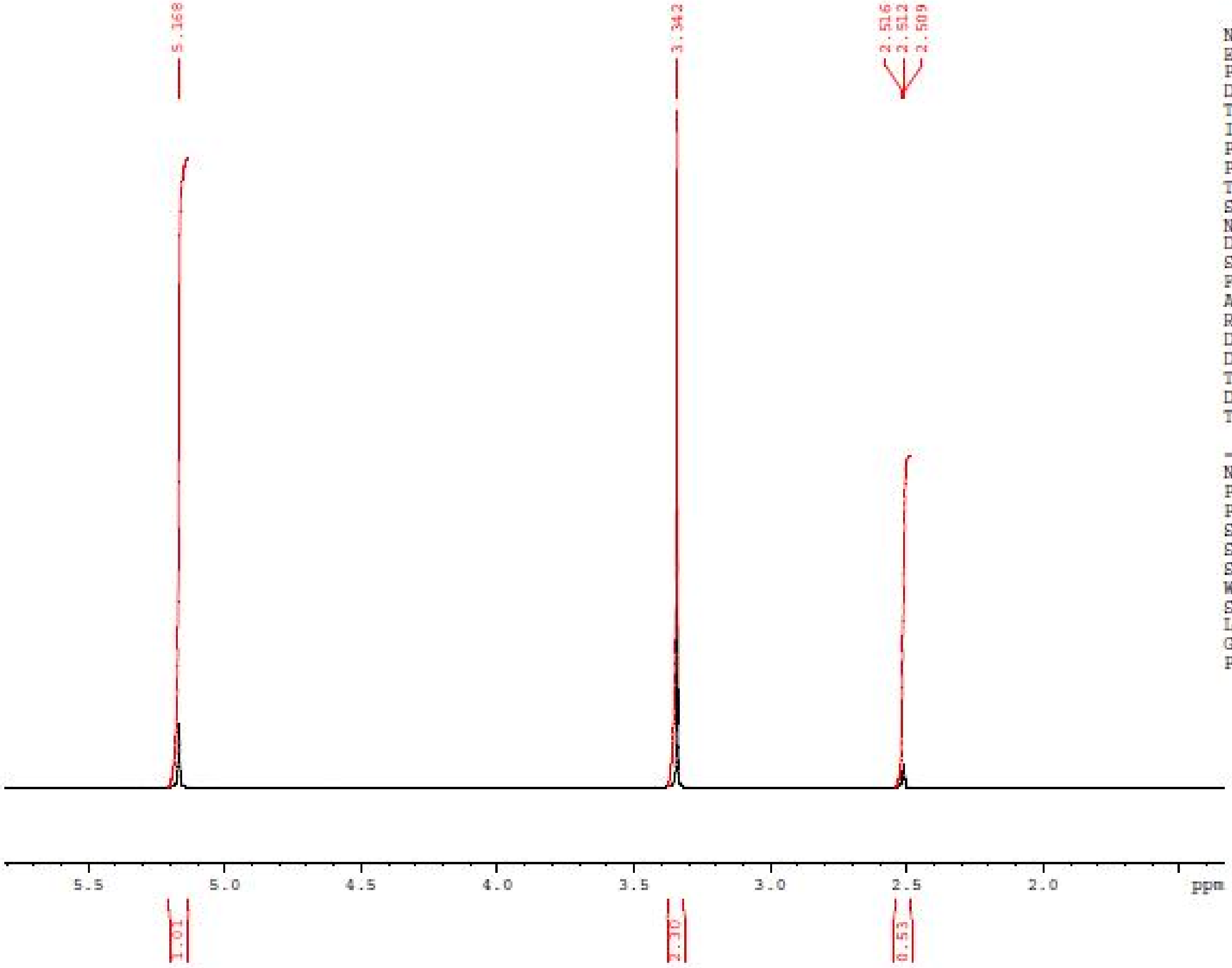

BRUKER

NAME	drhatem-5a
EXPNO	10
PROCNO	1
Date_	20130304
Time	16.39
INSTRUM	spect
PROBHD	5 mm PABBO BB-
PULPROG	zg30
TD	65536
SOLVENT	DMSO
NS	16
DS	2
SWH	10330.578 Hz
FIDRES	0.157632 Hz
AQ	3.1720407 sec
RG	128
DW	48.400 usec
DE	6.50 usec
TE	301.1 K
D1	1.00000000 sec
TD0	1

======== CHANNEL f1 ========

NUC1	1H
P1	14.70 usec
PL1	-1.00 dB
SFO1	500.1330885 MHz
SI	32768
SF	500.1300000 MHz
WDW	EM
SSB	0
LB	0.30 Hz
GB	0
PC	1.00

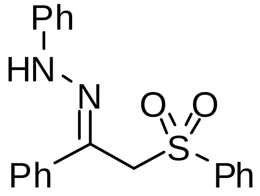

17a

3BO DMSO D:\\ mmj

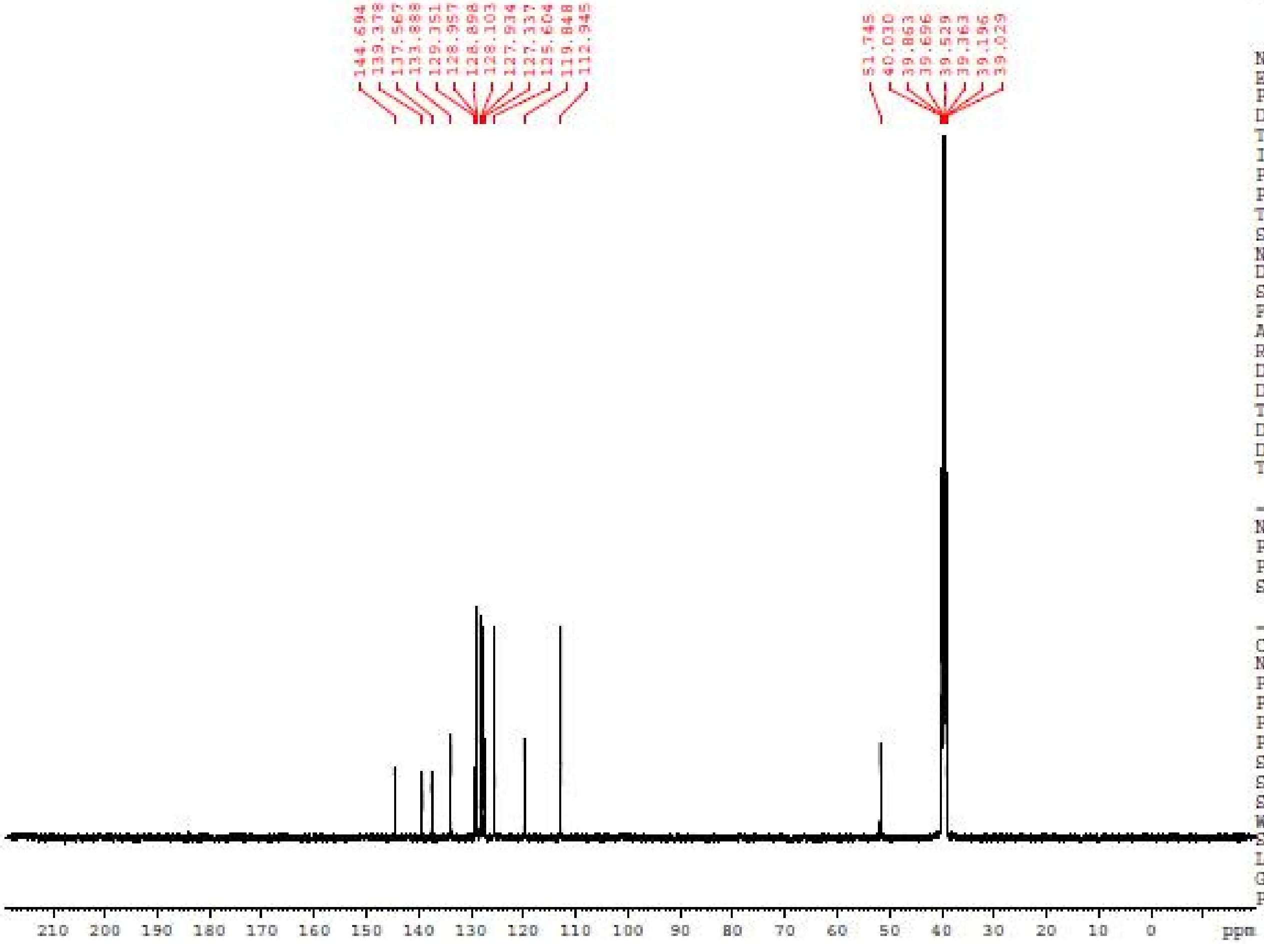

BRUKER

NAME drhatem-5a
EXPNO 11
PROCNO 1
Date_ 20130304
Time 18.29
INSTRUM spect
PROBHD 5 mm PABBO BB-
PULPROG zgpg30
TD 65536
SOLVENT DMSO
NS 2048
DS 4
SWH 30030.029 Hz
FIDRES 0.458222 Hz
AQ 1.0912410 sec
RG 20642.5
DW 16.650 usec
DE 6.50 usec
TE 302.0 K
D1 2.00000000 sec
D11 0.03000000 sec
TD0 1

======== CHANNEL f1 ========
NUC1 13C
P1 10.00 usec
PL1 -5.00 dB
SFO1 125.7703643 MHz

======== CHANNEL f2 ========
CPDPRG2 waltz16
NUC2 1H
PCPD2 80.00 usec
PL2 -1.00 dB
PL12 13.40 dB
PL13 16.40 dB
SFO2 500.1320005 MHz
SI 32768
SF 125.7578519 MHz
WDW EM
SSB 0
LB 3.00 Hz
GB 0
PC 1.40

3BO DMSO D:\\ mmj

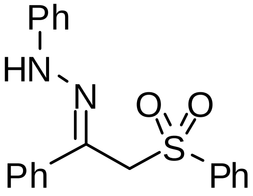

17a

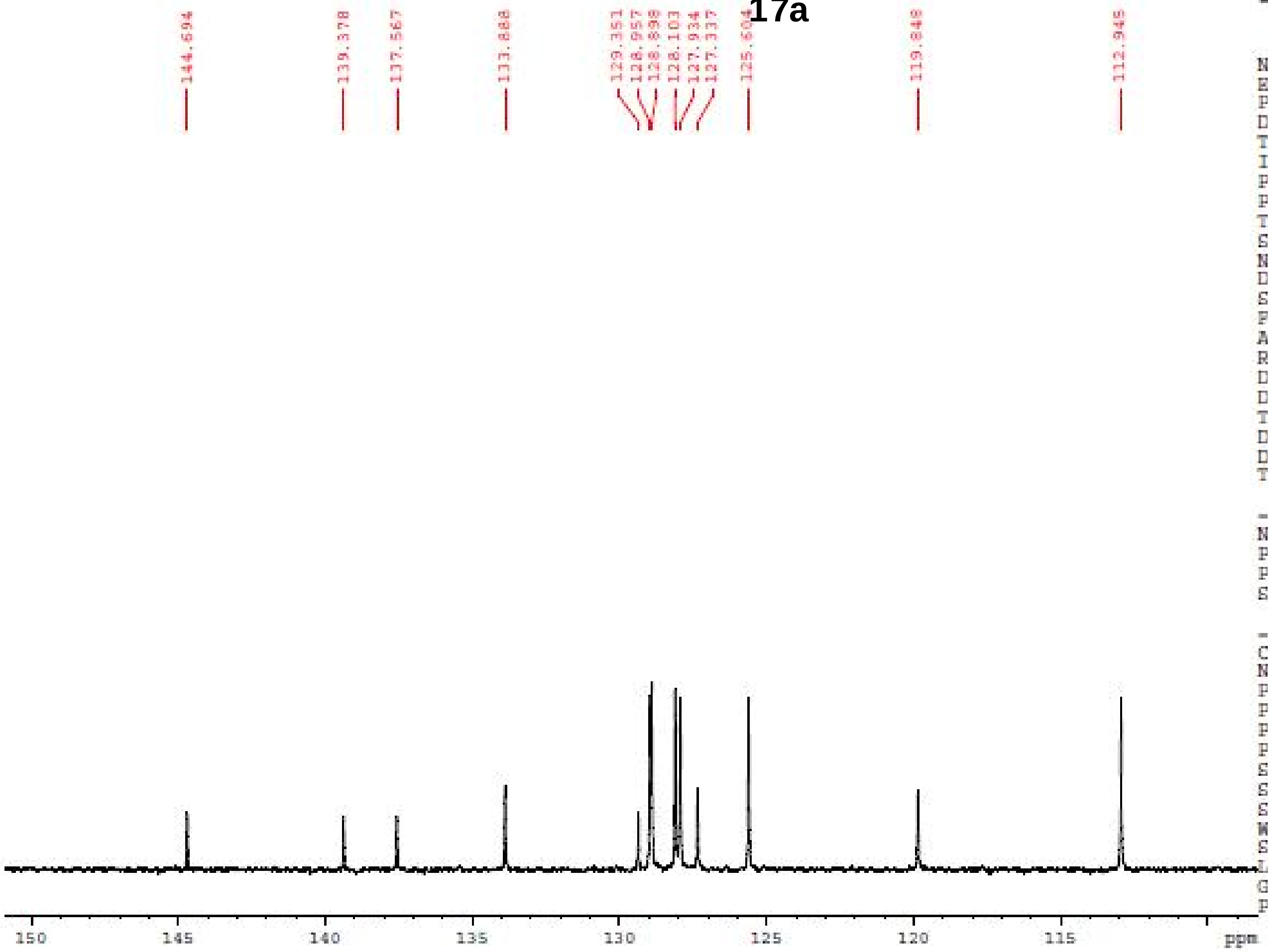

NAME	drhatem-5a
EXPNO	11
PROCNO	1
Date_	20130304
Time	18.29
INSTRUM	spect
PROBHD	5 mm PABBO BB-
PULPROG	zgpg30
TD	65536
SOLVENT	DMSO
NS	2048
DS	4
SWH	30030.029 Hz
FIDRES	0.458222 Hz
AQ	1.0912410 sec
RG	20642.5
DW	16.650 usec
DE	6.50 usec
TE	302.0 K
D1	2.00000000 sec
D11	0.03000000 sec
TD0	1

======== CHANNEL f1 ========

NUC1	13C
P1	10.00 usec
PL1	-5.00 dB
SFO1	125.7703643 MHz

======== CHANNEL f2 ========

CPDPRG2	waltz16
NUC2	1H
PCPD2	80.00 usec
PL2	-1.00 dB
PL12	13.40 dB
PL13	16.40 dB
SFO2	500.1320005 MHz
SI	32768
SF	125.7578519 MHz
WDW	EM
SSB	0
LB	3.00 Hz
GB	0
PC	1.40

3BO DMSO D:\\ mmj

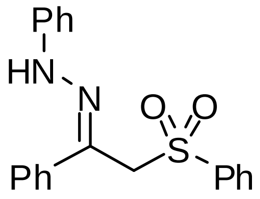

17a

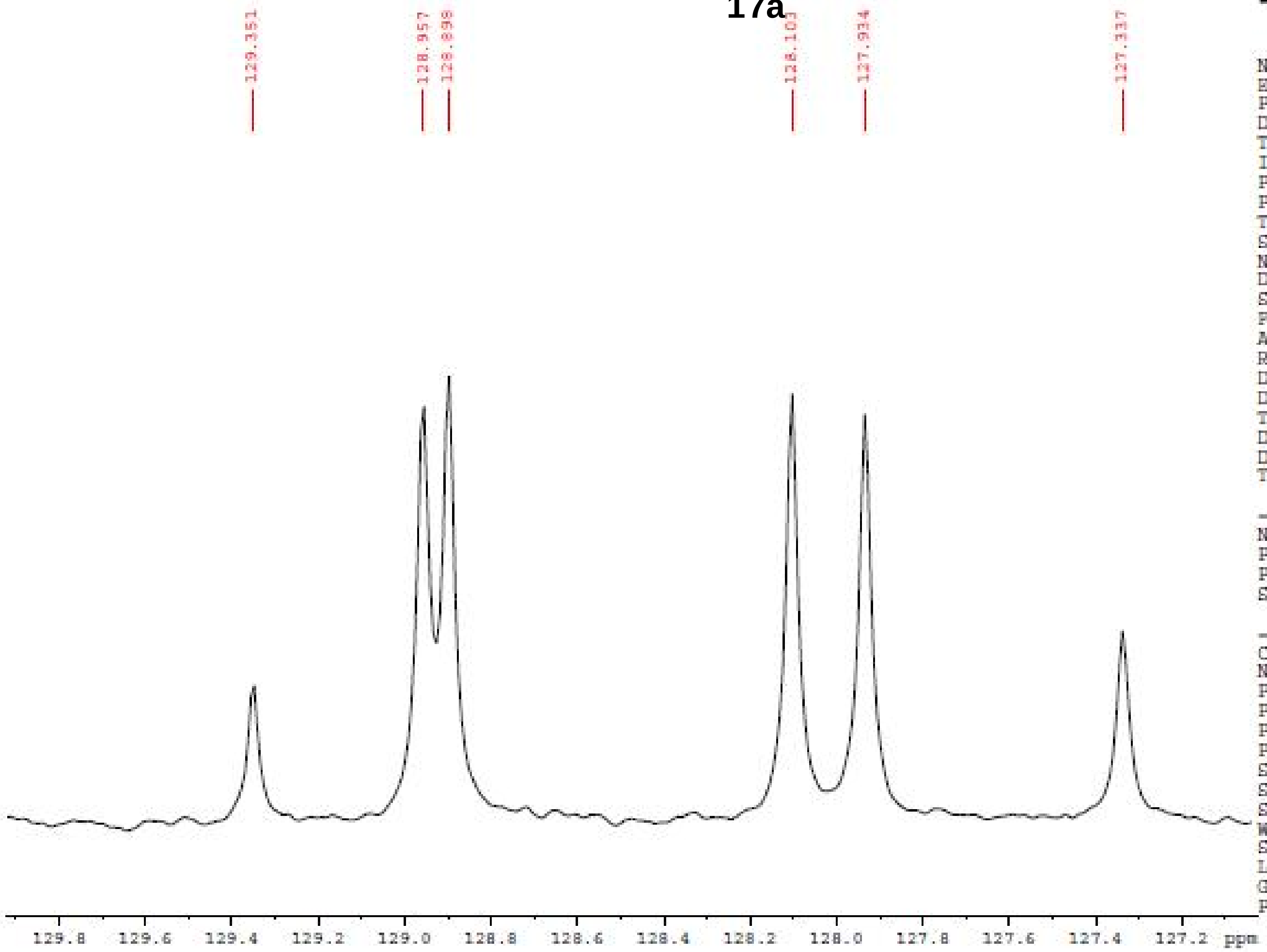

NAME	drhatem-5a
EXPNO	11
PROCNO	1
Date_	20130304
Time	18.29
INSTRUM	spect
PROBHD	5 mm PABBO BB-
PULPROG	zgpg30
TD	65536
SOLVENT	DMSO
NS	2048
DS	4
SWH	30030.029 Hz
FIDRES	0.458222 Hz
AQ	1.0912410 sec
RG	20642.5
DW	16.650 usec
DE	6.50 usec
TE	302.0 K
D1	2.00000000 sec
D11	0.03000000 sec
TD0	1

======== CHANNEL f1 ========

NUC1	13C
P1	10.00 usec
PL1	-5.00 dB
SFO1	125.7703643 MHz

======== CHANNEL f2 ========

CPDPRG2	waltz16
NUC2	1H
PCPD2	80.00 usec
PL2	-1.00 dB
PL12	13.40 dB
PL13	16.40 dB
SFO2	500.1320005 MHz
SI	32768
SF	125.7578519 MHz
WDW	EM
SSB	0
LB	3.00 Hz
GB	0
PC	1.40

-BBO DMSO D:\\ mi

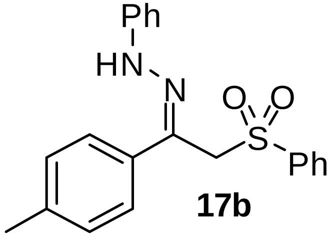

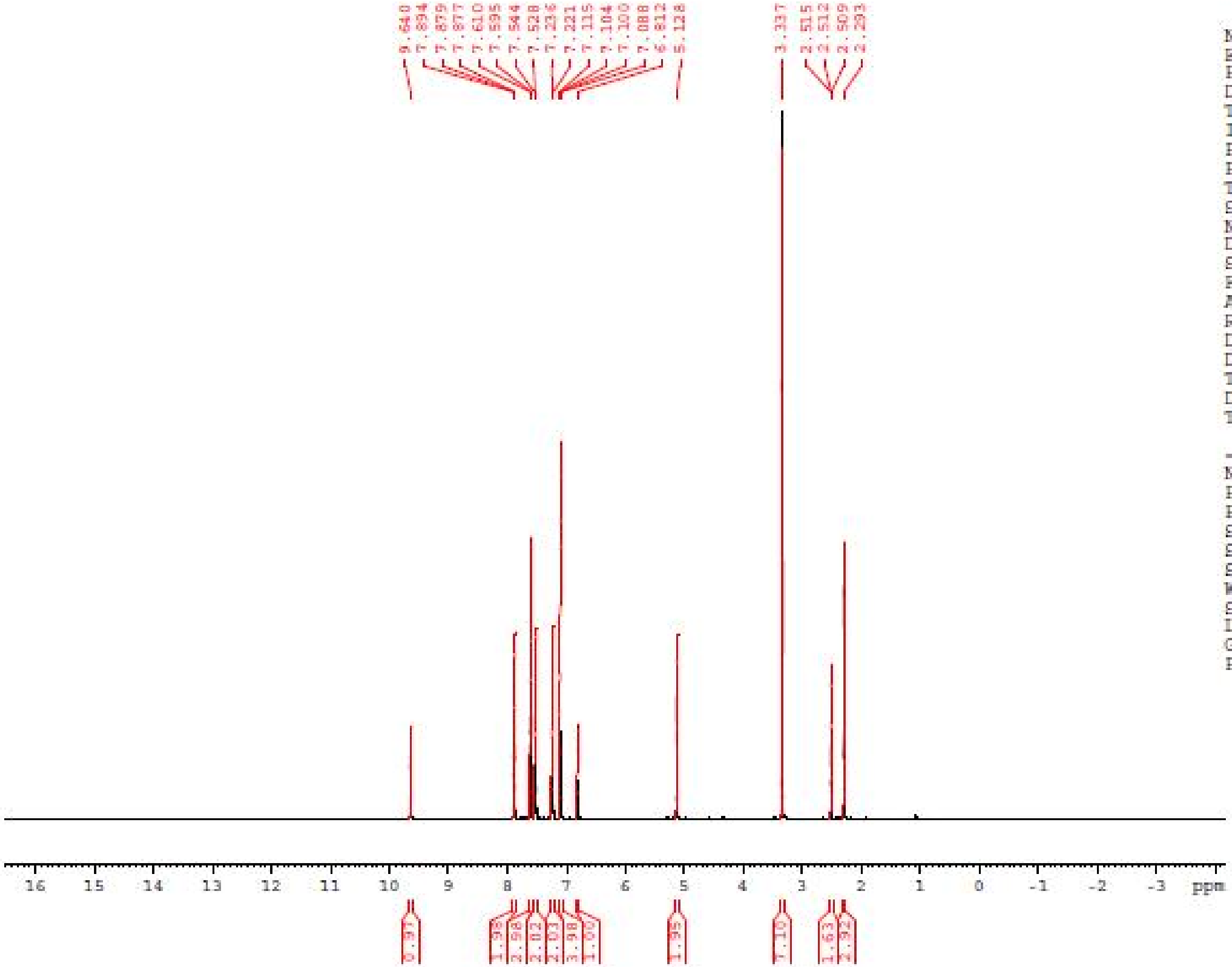

BRUKER

NAME	drhatem-5b
EXPNO	10
PROCNO	1
Date_	20130304
Time	14.45
INSTRUM	spect
PROBHD	5 mm PABBO BB-
PULPROG	zg30
TD	65536
SOLVENT	DMSO
NS	16
DS	2
SWH	10330.578 Hz
FIDRES	0.157632 Hz
AQ	3.1720407 sec
RG	128
DW	48.400 usec
DE	6.50 usec
TE	301.0 K
D1	1.00000000 sec
TD0	1

======== CHANNEL f1 ========

NUC1	1H
P1	14.70 usec
PL1	-1.00 dB
SFO1	500.1330885 MHz
SI	32768
SF	500.1300000 MHz
WDW	EM
SSB	0
LB	0.30 Hz
GB	0
PC	1.00

-BBO DMSO D:\\ m

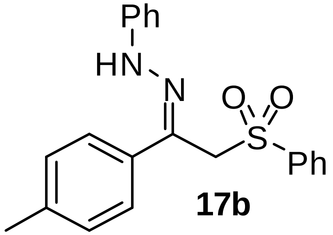

17b

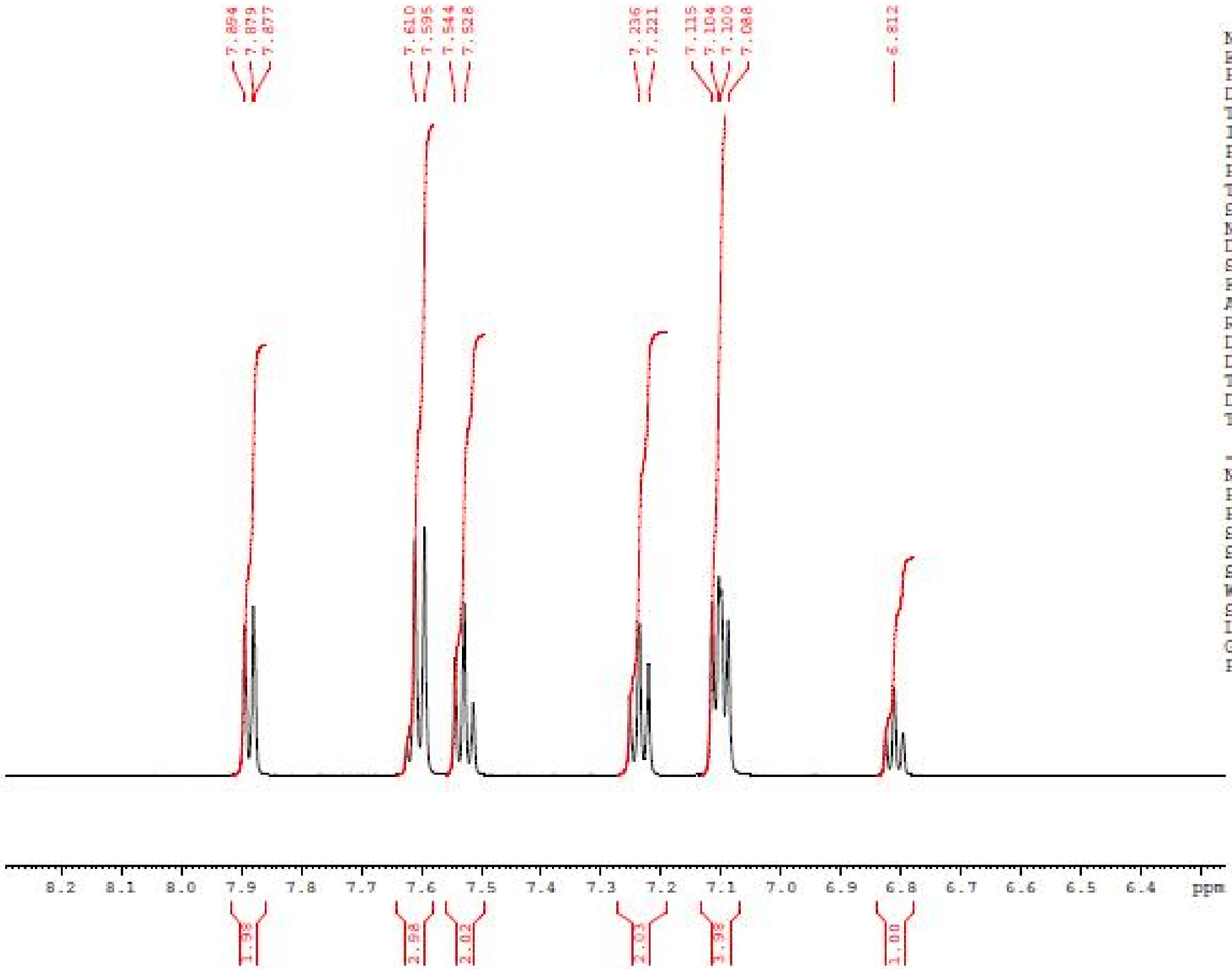

BRUKER

NAME	drhatem-5b
EXPNO	10
PROCNO	1
Date_	20130304
Time	14.45
INSTRUM	spect
PROBHD	5 mm PABBO BB-
PULPROG	zg30
TD	65536
SOLVENT	DMSO
NS	16
DS	2
SWH	10330.578 Hz
FIDRES	0.157632 Hz
AQ	3.1720407 sec
RG	128
DW	48.400 usec
DE	6.50 usec
TE	301.0 K
D1	1.00000000 sec
TD0	1

======== CHANNEL f1 ========

NUC1	1H
P1	14.70 usec
PL1	-1.00 dB
SFO1	500.1330885 MHz
SI	32768
SF	500.1300000 MHz
WDW	EM
SSB	0
LB	0.30 Hz
GB	0
PC	1.00

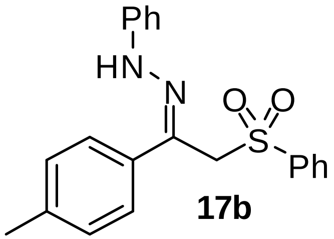

17b

-BBO DMSO D:\\ m

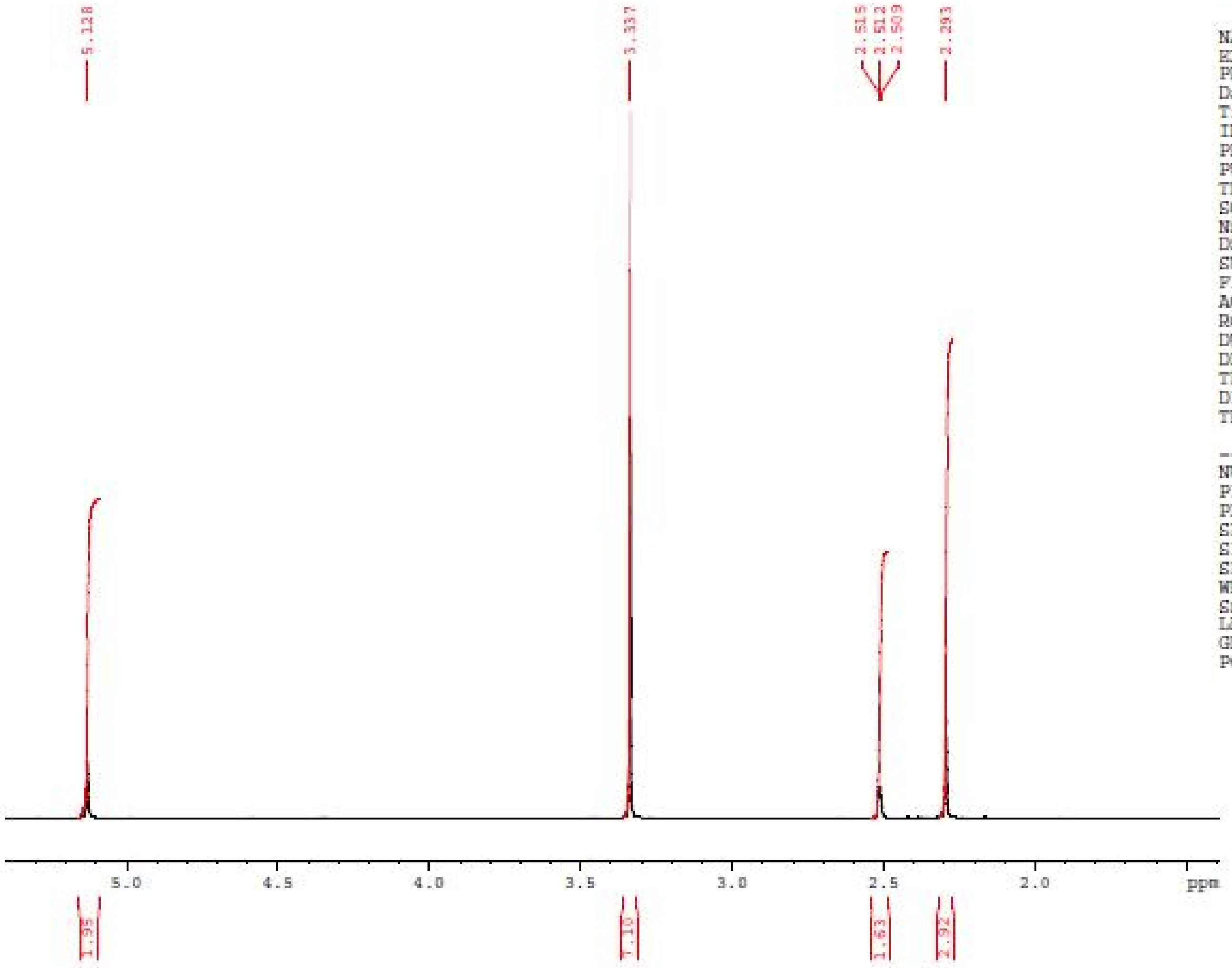

BRUKER

NAME	drhatem-5b	
EXPNO	10	
PROCNO	1	
Date_	20130304	
Time	14.45	
INSTRUM	spect	
PROBHD	5 mm PABBO BB-	
PULPROG	zg30	
TD	65536	
SOLVENT	DMSO	
NS	16	
DS	2	
SWH	10330.578	Hz
FIDRES	0.157632	Hz
AQ	3.1720407	sec
RG	128	
DW	48.400	usec
DE	6.50	usec
TE	301.0	K
D1	1.00000000	sec
TD0	1	
======== CHANNEL f1 ========		
NUC1	1H	
P1	14.70	usec
PL1	-1.00	dB
SFO1	500.1330885	MHz
SI	32768	
SF	500.1300000	MHz
WDW	EM	
SSB	0	
LB	0.30	Hz
GB	0	
PC	1.00	

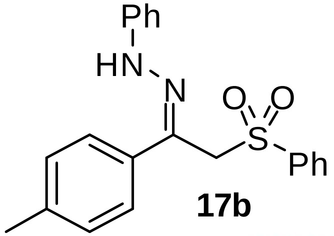

Ph
HN
N
O
O
S
Ph

17b

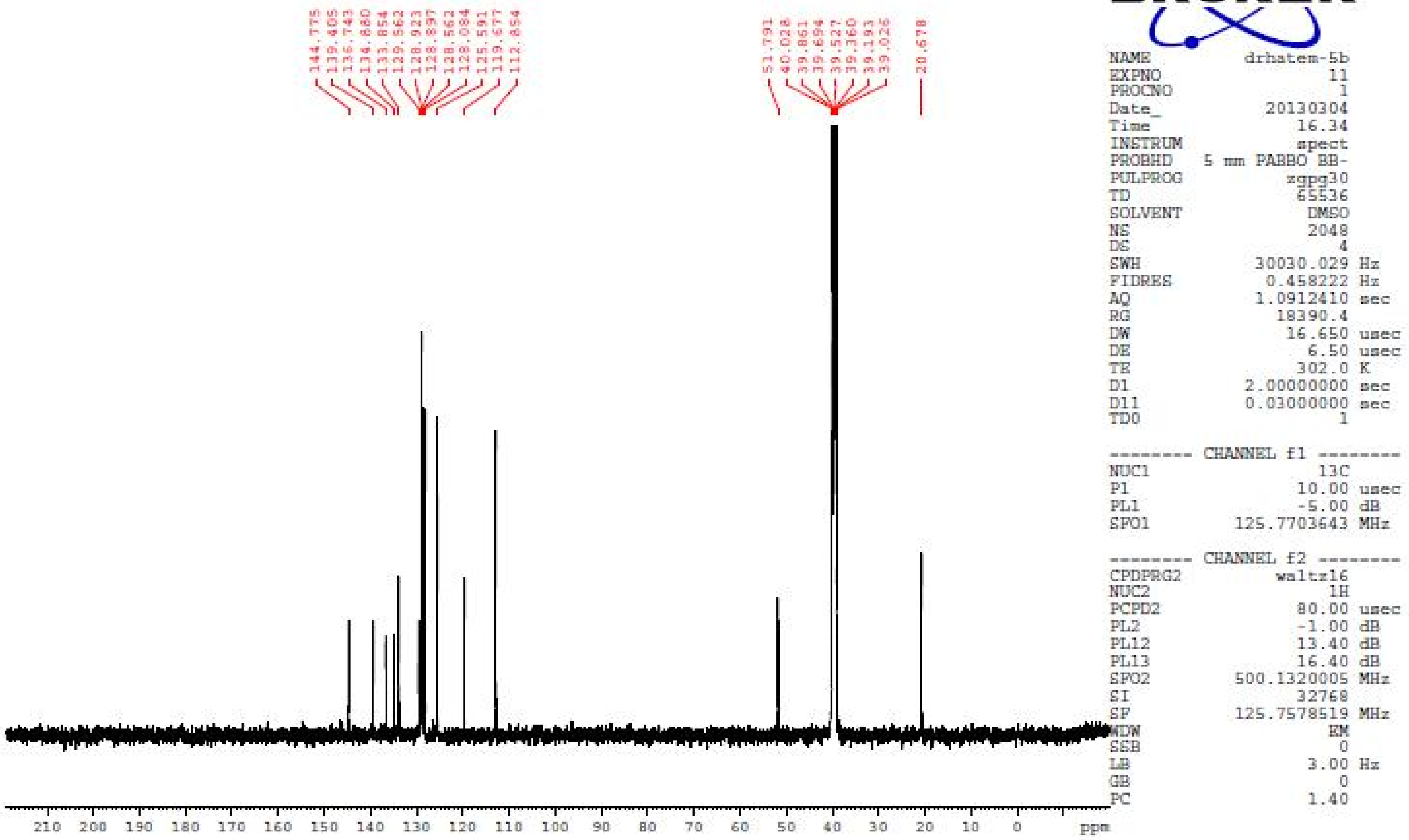

3BO DMSO D:\\ mmj
144.775
139.405
136.743
134.880
133.854
129.562
128.923
128.897
128.562
128.084
125.591
119.677
112.854
51.791
40.028
39.861
39.694
39.527
39.360
39.193
39.026
20.678
BRUKER
NAME drhatem-5b
EXPNO 11
PROCNO 1
Date_ 20130304
Time 16.34
INSTRUM spect
PROBHD 5 mm PABBO BB-
PULPROG zgpg30
TD 65536
SOLVENT DMSO
NS 2048
DS 4
SWH 30030.029 Hz
FIDRES 0.458222 Hz
AQ 1.0912410 sec
RG 18390.4
DW 16.650 usec
DE 6.50 usec
TE 302.0 K
D1 2.00000000 sec
D11 0.03000000 sec
TD0 1
======== CHANNEL f1 ========
NUC1 13C
P1 10.00 usec
PL1 -5.00 dB
SFO1 125.7703643 MHz
======== CHANNEL f2 ========
CPDPRG2 waltz16
NUC2 1H
PCPD2 80.00 usec
PL2 -1.00 dB
PL12 13.40 dB
PL13 16.40 dB
SFO2 500.1320005 MHz
SI 32768
SF 125.7578519 MHz
WDW EM
SSB 0
LB 3.00 Hz
GB 0
PC 1.40
210 200 190 180 170 160 150 140 130 120 110 100 90 80 70 60 50 40 30 20 10 0 ppm

3BO DMSO D:\\ mmj

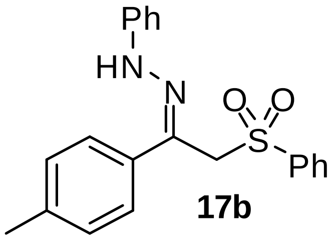

17b

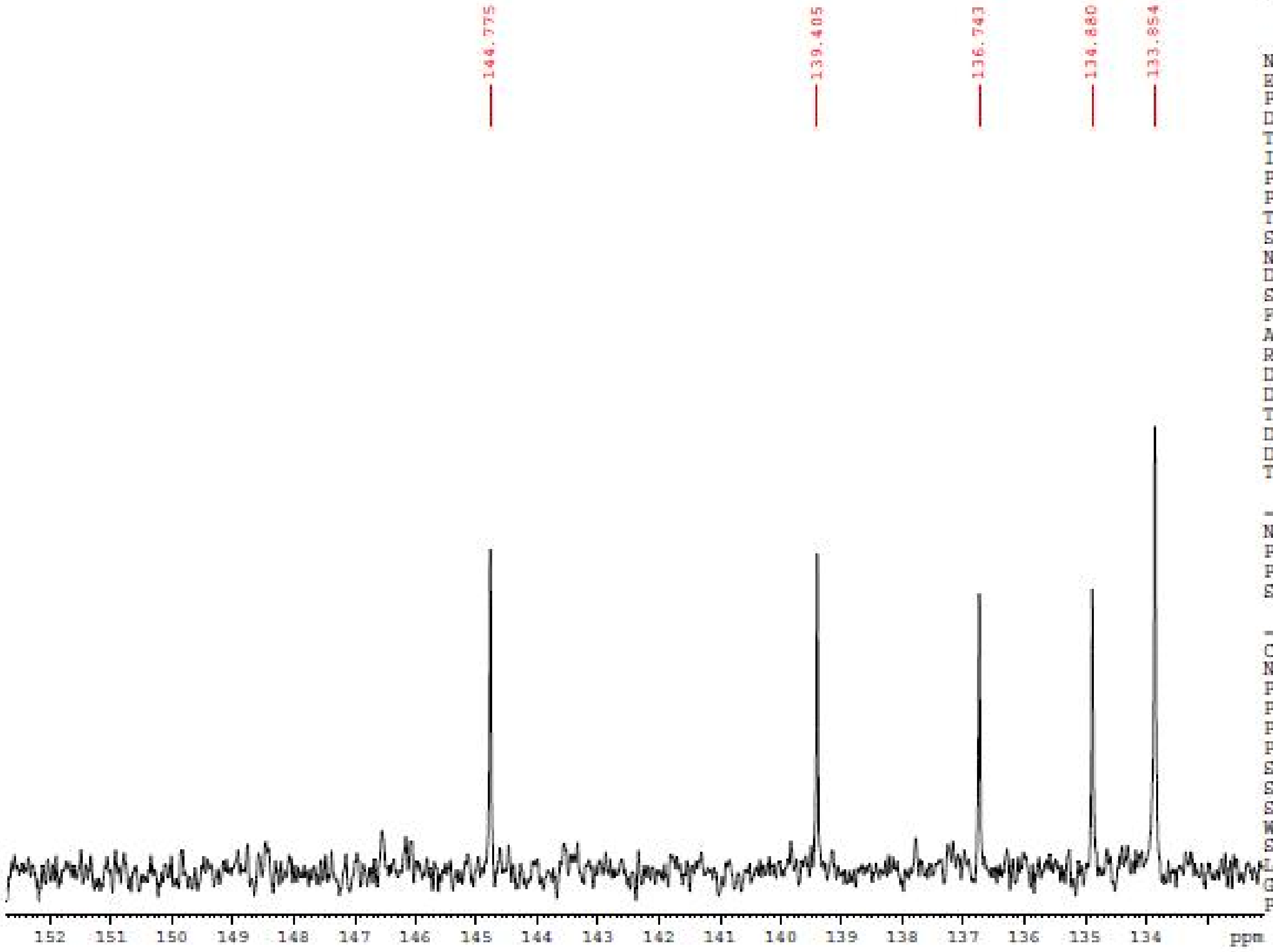

NAME	drhatem-5b	
EXPNO	11	
PROCNO	1	
Date_	20130304	
Time	16.34	
INSTRUM	spect	
PROBHD	5 mm PABBO BB-	
PULPROG	zgpg30	
TD	65536	
SOLVENT	DMSO	
NS	2048	
DS	4	
SWH	30030.029	Hz
FIDRES	0.458222	Hz
AQ	1.0912410	sec
RG	18390.4	
DW	16.650	usec
DE	6.50	usec
TE	302.0	K
D1	2.00000000	sec
D11	0.03000000	sec
TD0	1	

======== CHANNEL f1 ========

NUC1	13C	
P1	10.00	usec
PL1	-5.00	dB
SFO1	125.7703643	MHz

======== CHANNEL f2 ========

CPDPRG2	waltz16	
NUC2	1H	
PCPD2	80.00	usec
PL2	-1.00	dB
PL12	13.40	dB
PL13	16.40	dB
SFO2	500.1320005	MHz
SI	32768	
SF	125.7578519	MHz
WDW	EM	
SSB	0	
LB	3.00	Hz
GB	0	
PC	1.40	

3BO DMSO D:\\ mmj

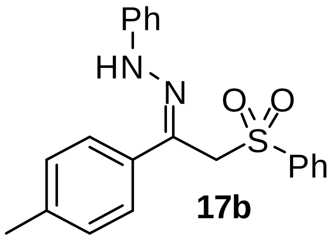

17b

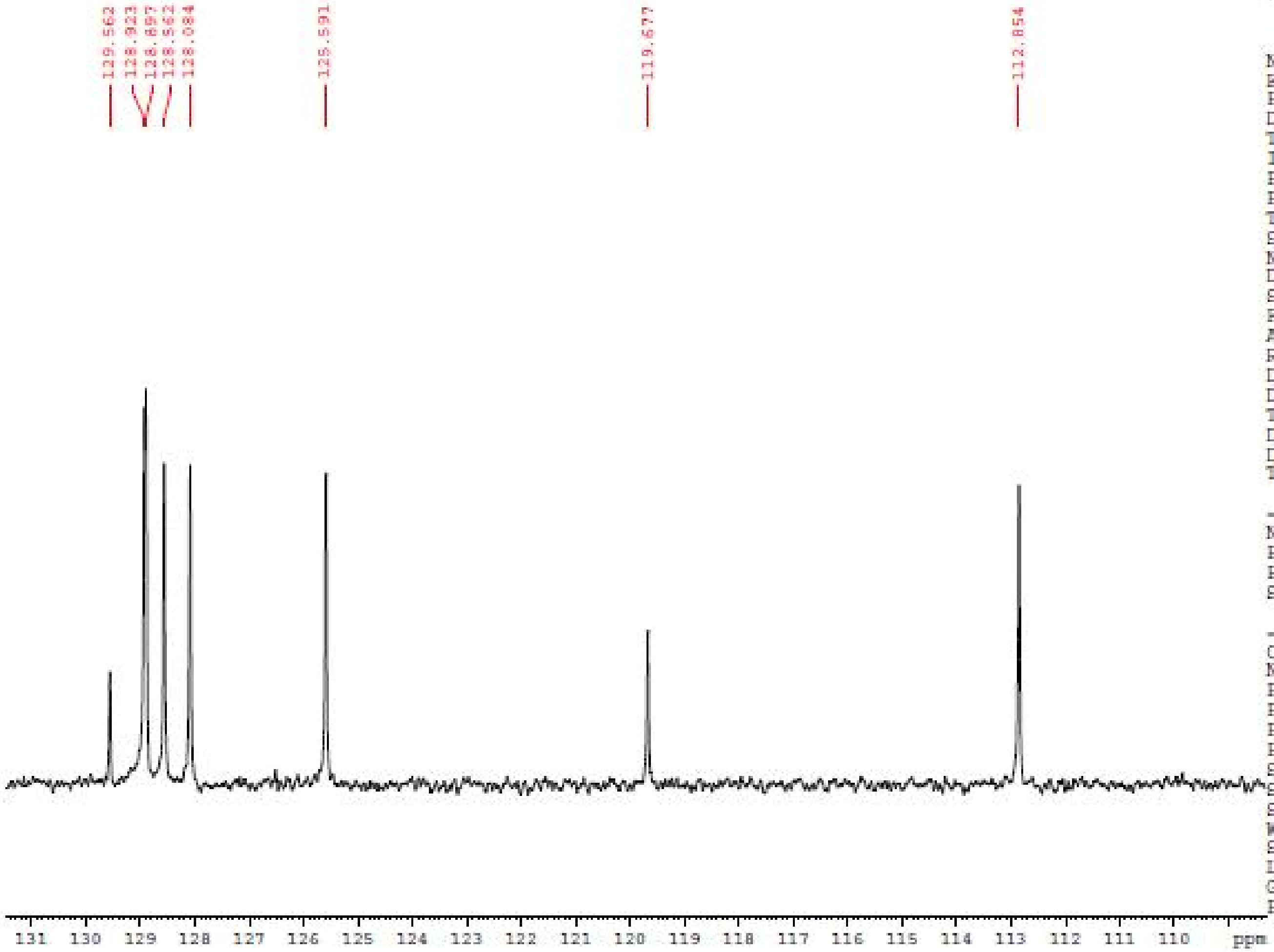

NAME	drhatem-5b
EXPNO	11
PROCNO	1
Date_	20130304
Time	16.34
INSTRUM	spect
PROBHD	5 mm PABBO BB-
PULPROG	zgpg30
TD	65536
SOLVENT	DMSO
NS	2048
DS	4
SWH	30030.029 Hz
FIDRES	0.458222 Hz
AQ	1.0912410 sec
RG	18390.4
DW	16.650 usec
DE	6.50 usec
TE	302.0 K
D1	2.00000000 sec
D11	0.03000000 sec
TD0	1

======== CHANNEL f1 ========

NUC1	13C
P1	10.00 usec
PL1	-5.00 dB
SFO1	125.7703643 MHz

======== CHANNEL f2 ========

CPDPRG2	waltz16
NUC2	1H
PCPD2	80.00 usec
PL2	-1.00 dB
PL12	13.40 dB
PL13	16.40 dB
SFO2	500.1320005 MHz
SI	32768
SF	125.7578519 MHz
WDW	EM
SSB	0
LB	3.00 Hz
GB	0
PC	1.40
